# Multi‐Trait GWAS Analyses of Blood Pressure Traits Provide Limited Gains in Locus Discovery or Polygenic Risk Score Performance Compared to Single‐Trait GWAS

**DOI:** 10.1002/gepi.70062

**Published:** 2026-10-22

**Authors:** Yujia Ma, Anna D. Argoty‐Pantoja, Tian Xie, Michael R. Barnes, Patricia B. Munroe, Claudia P. Cabrera, Harold Snieder, Helen R. Warren, UMCG Genetics Lifelines Initiative (UGLI) group author: LifeLines Cohort Study, Raul Aguirre‐Gamboa, Patrick Deelen, Lude Franke, Jan A. Kuivenhoven, Esteban A. Lopera Maya, Ilja M. Nolte, Serena Sanna, Harold Snieder, Morris A. Swertz, Peter M. Visscher, Judith M. Vonk, Cisca Wijmenga, Naomi Wray

**Affiliations:** ^1^ Department of Epidemiology University of Groningen, University Medical Center Groningen Groningen the Netherlands; ^2^ Department of Non‐communicable Chronic Disease Control and Prevention Beijing Center for Disease Prevention and Control Beijing China; ^3^ Guangzhou National Laboratory Guangdong Province Guangzhou China; ^4^ William Harvey Research Institute, Barts and The London Faculty of Medicine and Dentistry Queen Mary University of London London UK; ^5^ National Institute for Health and Care Research, Barts Cardiovascular Biomedical Research Centre Queen Mary University of London London UK; ^6^ Department of Genetics University of Groningen, University Medical Center Groningen Groningen The Netherlands; ^7^ Department of Pediatrics University of Groningen, University Medical Center Groningen Groningen The Netherlands; ^8^ Institute for Molecular Bioscience The University of Queensland Brisbane Queensland Australia

**Keywords:** blood pressure, GenomicSEM, hypertension, multi‐trait GWAS

## Abstract

High blood pressure (BP) represents a major cause of life‐threatening cardiovascular conditions. GWAS have identified >2000 BP‐associated signals. BP‐GWAS usually analyze each BP trait individually. However, multi‐trait GWAS may yield novel loci and improve polygenic risk scores (PRS) prediction. We explored whether multi‐trait BP‐GWAS can: (i) find novel loci undetected by single‐trait GWAS; (ii) increase heritability estimates; (iii) improve PRS prediction of hypertension within the Lifelines Cohort Study. We investigate the utility of multi‐trait GWAS approaches of correlated BP traits (systolic BP, diastolic BP, pulse pressure). We tested two multi‐trait methods inputting results from the latest *N* > 1 million single‐trait BP‐GWAS: (a) Multi‐Trait Analysis of GWAS (MTAG); (b) genomic structural equation modeling (GenomicSEM); and compared results with single‐trait results. We only identified one novel locus from multi‐trait GWAS: *ZNF268*. Neither heritability estimates nor PRS performance improved using multi‐trait GWAS. In this scenario of complete sample overlap across BP‐GWAS, we observed little gain in results from multi‐trait BP‐GWAS over single‐trait BP‐GWAS. Our findings quantify the limited benefit of multi‐trait methods under this specific setting. Future BP genetic discoveries are more likely to come from larger GWAS adding new cohort data. However, conclusions may differ for other traits, depending on trait‐specific genetic architecture.

## 1 Introduction

High blood pressure (BP) is a major modifiable risk factor of cardiovascular and circulatory diseases. When unnoticed and uncontrolled it can lead to lifelong disability or fatal consequences. The combination of technological and methodological advances, together with increased sample sizes in genome‐wide association studies (GWAS), has led to the discovery of over 2000 BP loci (Evangelou et al. 2018; Giri et al. 2019; Hoffmann et al. 2017; Keaton et al. 2024; Surendran et al. 2020; Warren et al. 2017). BP‐GWAS analyses have reached sample sizes of more than one million individuals and yet BP loci still only explain approximately one‐third of the genetic contribution, still leaving much of the variation unaccounted for (Keaton et al. 2024).

Most BP GWAS have previously focused on single‐trait analyses (univariate analysis), with systolic BP (SBP), diastolic BP (DBP), and pulse pressure (PP) being analyzed independently but their loci reported jointly. However, this misses the opportunity of gaining power from assessing correlated traits in a multivariate approach, which these correlated BP traits would be well suited to. For example, a multivariate analysis of summary statistics of correlated traits (CPASSOC) (Li and Zhu 2017) applied to SBP, DBP, and Hypertension status (HTN) in *N* = 29,378 individuals of African descent from the COGENT study in 2015 led to the discovery of four loci which were not reported by the original single‐trait analyses (Zhu et al. 2015).

Biobanks and cohort studies typically collect a large number of phenotypes, allowing analyses of multiple correlated traits in thousands of samples. A variety of multi‐trait GWAS approaches has been developed in recent years (Hackinger and Zeggini 2017), e.g., metaCCA (Cichonska et al. 2016); Cross Phenotype Meta‐Analysis (Cotsapas et al. 2011); JASS (Julienne et al. 2020); heritability‐informed power optimization (HIPO) (Qi and Chatterjee 2018); MTAR (Guo and Wu 2019); multi‐trait mixed model (MTMM) (Korte et al. 2012); Multivariate Omnibus Statistical Test (MOSTest) (van der Meer et al. 2020). However, most methodologies either need individual‐level genotypic and phenotypic data, or only provide one‐single association result output across all analyzed traits.

Therefore, we have selected two different multi‐trait approaches to compare: (a) Multi‐Trait Analysis of GWAS (MTAG); (b) genomic structural equation modeling (GenomicSEM), as they both only require summary statistics as input. An advantage of MTAG is that the output still provides separate summary statistics per trait, enabling easier comparison to the original single‐trait results. In contrast, as GenomicSEM provides one combined output, it may have more beneficial implementation for creating one‐single PRS representing the overall BP genetic profile to use more simply for the prediction of hypertension. BP studies are challenging with the complexity of multiple different BP traits to analyze, such as SBP, DBP, and PP. When previously performing prediction analyses of BP‐PRS from BP‐GWAS projects, we have considered different options: (i) single‐trait prediction, for example, just testing the SBP‐PRS for prediction of hypertension; (ii) calculating one single average BP‐PRS variable, where all SNPs in both the SBP‐PRS and DBP‐PRS are included and their weights are taken as the mean of their SBP‐PRS and DBP‐PRS weights; (iii) multivariate BP‐PRS prediction models including both SBP‐PRS and DBP‐PRS. But neither option seems ideal analytically, and we did not believe them to be optimal approaches. In order to simplify any future applications and analyses of BP‐PRS, we were therefore keen to investigate more practical alternatives with one‐single PRS variable, which a GenomicSEM model would enable.

It is timely, following the latest BP‐GWAS analyses in *N* > 1 million individuals, to investigate the utility of multi‐trait BP‐GWAS analyses in a setting of complete sample overlap across the underlying GWAS and compare the results of single‐trait vs. multi‐trait GWAS, in order to: (i) search for any potential novel loci from multi‐trait GWAS that have been previously undetected from single‐trait GWAS; (ii) calculate if multi‐trait GWAS yield any increases in heritability estimates, and (iii) investigate the performance of PRS derived from multi‐trait GWAS for the prediction of hypertension within an independent, external cohort, Lifelines.

## 2 Methods

We applied two multivariate approaches (GenomicSEM and MTAG) to the latest GWAS on BP conducted in 1,028,980 European individuals (Keaton et al. 2024). The discovery study analyzed ~7.5 million imputed single‐nucleotide polymorphisms (SNPs) with minor allele frequency (MAF) ≥ 1%. Importantly, we note that all three GWAS for SBP, DBP, and PP are based on exactly the same samples, that is, in a scenario of complete sample overlap. We performed three different two‐trait models: SBP‐DBP, SBP‐PP, and DBP‐PP. As PP is a derived variable calculated as the difference between SBP and DBP, we did not perform a multivariate SBP‐DBP‐PP model, because including PP would introduce high collinearity, which could compromise the accuracy of the estimates and the reliability of the results.

GenomicSEM is a structural equation modeling approach used to assess the shared genetic architecture of complex traits by leveraging summary statistics from univariate GWASs (Grotzinger et al. 2019). This method applies structural equation modeling to genetic covariance matrices derived from GWAS summary statistics through a two‐stage estimation process (Grotzinger et al. 2019). In the first stage, a genetic covariance matrix and a sampling covariance matrix are estimated, typically using Linkage Disequilibrium Score Regression (LDSC). In the second stage, a user‐defined SEM is fit to the genetic covariance matrix, incorporating equality constraints on the factor loadings for each two‐indicator latent factor to ensure model identification. The user‐defined SEM GWAS for the latent factor then considers the effects of genetic variants on the shared heritability of related traits. Parameters and their standard errors were estimated using weighted least squares estimation (Grotzinger et al. 2019). The GenomicSEM analysis was conducted using the *GenomicSEM* R package (Grotzinger et al. 2019). We obtained results as one GenomicSEM overall latent factor output, according to each of the three different two‐trait models: GenomicSEM(SBP‐DBP), GenomicSEM(SBP‐PP), and GenomicSEM(DBP‐PP),

MTAG is a generalization of inverse‐variance‐weighted meta‐analysis, which allows us to analyze the summary statistics of multiple related traits and reports a trait‐specific estimate for each SNP (Turley et al. 2018). Moreover, it accounts for sample overlap using the bivariate LDSC approach. The MTAG analysis was performed using MTAG (v1.0.8) on Python 2.7.5. We obtained six MTAG outputs, one for each separate BP trait from the three different two‐trait analyses: SBP‐MTAG(SBP‐DBP), SBP‐MTAG(SBP‐PP), DBP‐MTAG(SBP‐DBP), DBP‐MTAG(DBP‐PP), PP‐MTAG(SBP‐PP), and PP‐MTAG(DBP‐PP).

The same analysis design was applied for GenomicSEM and MTAG. The HLA region (25M‐35M) was excluded, and only shared SNPs between MTAG and GenomicSEM were used for further analysis (*n* = 7,124,044).

### 2.1 Part (1) Comparison Between GWAS, MTAG, and GenomicSEM Results

We compared all pairs of results across each of the three different two‐trait model analysis outputs from multi‐trait GWAS: three overall latent factors from GenomicSEM and six trait‐specific outputs from MTAG. To evaluate the performance of our approaches, we compared the results between the two different multi‐trait approaches: GenomicSEM vs MTAG, as well as comparing the multi‐trait analysis results with those from the latest single‐trait GWAS (SBP, DBP, and PP) (Keaton et al. 2024), where all summary statistics were restricted to the common set of SNPs (*n* = 7,124,044). Across all models, we compared: the number of significant associations (clumps); beta coefficient effect estimates, *p*‐values, and genetic correlations.

#### 2.1.1 Significant Associations (Number of Clumps)

We used *clump* in Plink v1.9 to identify and count the number of independent signals from GenomicSEM and MTAG results at the genome‐wide significance threshold (*p* < 5 × 10^−8^) within 500 kb and an LD *r* ^2^ threshold of 0.1.

#### 2.1.2 Beta Coefficients and *p*‐value Comparison

We generated scatter plots comparing beta coefficients and *p*‐values for all SNPs across all models. In total, we generated 54 pairwise comparison plots. Additionally, we estimated the Pearson correlation coefficient (*r*) for beta coefficients and *p*‐values in each pairwise plot.

#### 2.1.3 Genetic correlation

We estimated genetic correlations (*r*_g_) between traits, using the summary statistics from each method through LDSC Regression by the built‐in *ldsc* function in the R package *GenomicSEM* (Grotzinger et al. 2019). This analysis was performed with European LD scores derived from 1000 Genomes and HapMap3 SNPs.

### 2.2 Part (2) Novel Loci Discovery

We ran each of the MTAG and GenomicSEM summary statistics outputs through a novel locus discovery pipeline, in order to search for any potential novel BP loci, that have not been previously published. For consistency, we used the same novel locus discovery pipeline that we had previously used within the BP‐GWAS (Keaton et al. 2024). We used the same split of the SNP dataset to partition each GWAS summary statistics dataset into “known” vs. “unknown” subsets, according to the augmented genomic regions around the same list of 3800 published BP SNPs that had been used as the “known” BP SNP list. Focusing then only on the “unknown” subset of each multi‐trait GWAS summary statistics dataset, we filtered SNPs according to the same significance reporting criteria used previously (Keaton et al. 2024):

i. genome‐wide significant SNPs with multi‐trait GWAS *p*‐value < 5 × 10^−8^;
ii. consistent direction of effect in all available studies from the original BP‐single‐trait GWAS meta‐analysis (for MTAG, using the same trait‐specific BP‐GWAS; for GenomicSEM, using the BP‐GWAS of the first analyzed trait from the 2‐trait BP model);
iii. heterogeneity *p*‐value > 1 × 10^−4^ from the corresponding single‐trait GWAS.

For each GWAS summary statistics dataset, after ranking all unique significant SNPs by *p*‐value significance, we clumped all SNPs into 1 MB loci by distance, using PLINK, so that no pairs of SNPs are within 500 kb of each other, identifying the lead sentinel SNP at each locus. All lead sentinel SNPs from each GWAS summary statistics dataset were then checked to be novel, compared to any published BP SNPs, according to the list of 3800 published SNPs from Sup Table 6 of the original study (Keaton et al. 2024) and the 113 novel loci & 267 secondary SNPs; confirming that any of our novel loci sentinel SNPs are: (a) > 500 kb away from, and (b) not in LD (*r* ^2^ < 0.1) with any published SNPs.

### 2.3 Part (3) LDSC Approach for Determination of Polygenicity and Heritability Estimation

We applied LDSC to each of the three single‐trait GWAS results (SBP, DBP, and PP) as well as nine multi‐trait GWAS results (three from GenomicSEM and six from MTAG) in order to compare their genomic inflation factors and their *h* ^2^ estimates of SNP‐based heritability (Bulik‐Sullivan et al. 2015). For GenomicSEM, we used the smallest sample size among the three phenotypes from the latest GWAS as the final sample size for latent factors. This was due to the fact that the effective sample size calculated by GenomicSEM was not on the same scale as the sample size in the BP‐ GWAS, which significantly reduced comparability.

### 2.4 Part (4) Polygenic Risk Scores and Hypertension Risk Prediction

Data from 10,210 participants in the Lifelines Cohort Study (Lifelines) were used to calculate polygenic risk scores (PRS), estimate the variance explained by PRS, and evaluate the association between PRS and hypertension. These are exactly the same samples that were also used in the PRS analyses from the single‐trait BP‐GWAS (Keaton et al. 2024), for consistent comparison. Lifelines is a multi‐disciplinary prospective population‐based cohort study examining, in a unique three‐generation design, the health and health‐related behaviors of 167,729 persons living in the North of the Netherlands. It employs a broad range of investigative procedures in assessing the biomedical, socio‐demographic, behavioral, physical, and psychological factors which contribute to the health and disease of the general population, with a special focus on multi‐morbidity and complex genetics (Sijtsma et al. 2022).

#### 2.4.1 PRS and Variance Explained

We leveraged five sets of summary statistics to generate corresponding PRSs using SBayesRC (Zheng et al. 2024): (1) GWAS‐SBP, (2) GWAS‐DBP, (3) GenomicSEM (SBP‐DBP), (4) MTAG‐SBP (SBP‐DBP), and 5) MTAG‐DBP (SBP‐DBP). Each trait‐specific PRS was calculated by multiplying the risk allele dosage of each SNP by its effect size and then summing across all SNPs. This Bayesian PRS method incorporates functional genomic annotations to enhance polygenic prediction by refining SNP effect estimates, and was the same approach used to generate the optimal BP‐PRS from the original single‐trait BP‐GWAS (Keaton et al. 2024). We then calculated the correlation between the five PRSs using the Pearson correlation coefficient (*r*).

We estimated the variance explained of each PRS for SBP, DBP, and hypertension, following the same estimation of a previous study (Keaton et al. 2024). Hypertension was defined as SBP ≥ 140 mmHg, or DBP ≥ 90 mmHg or self‐reported anti‐hypertensive medication use. For SBP and DBP, we used medication‐adjusted BP traits and built three linear regression models: (1) a model to estimate the residuals for each trait (BP ~ sex + age + age^2^ + BMI), (2) a model incorporating the top 10 principal components (PCs), where the outcome was the residuals from the first model (Residuals ~ 10 PCs), and (3) a model including both the 10 PCs and the PRS (Residuals ~ 10 PCs + PRS). The variance explained was estimated as the difference in the adjusted *R* ^2^ between the third and the second model. For hypertension, we built two logistic regression models: (1) a model adjusted for sex, age, age^2^, BMI, and the top 10 PCs, and (2) a model including the same variables plus the PRS. The variance explained was estimated as the difference in McFadden's *R* ^2^ between the two models.

#### 2.4.2 Association of PRSs Deciles With Hypertension

We evaluated the association between deciles of each PRS and hypertension using logistic regression models incorporating different PRS approaches:

- PRS from GenomicSEM: Since only one PRS was available, it was used directly in the analysis.
- PRSs from MTAG and GWAS: Since separate PRSs were available for SBP and DBP, we considered both the joint effect and the average effect in hypertension analyses:
  ◦ The joint *β* was estimated as the sum of *β* for SBP and *β* for DBP, and the joint standard error (SE) was calculated as the square root of the sum of the squared SEs of SBP and DBP plus twice the covariance (cov):
$\sqrt{{\left({\mathrm{SE}}_{\mathrm{SBP}}\right)}^{2}+{\left({\mathrm{SE}}_{\mathrm{DBP}}\right)}^{2}+2\;.\text{cov}}$
  ◦ The average effect was computed as the mean of the SBP PRS and DBP PRS: (SBP PRS + DBP PRS)/2
- We tested two model adjustments: a base model adjusted for sex only and a full model adjusted for age, age^2^, sex, BMI, and the top 10 PCs. Additionally, we examined models using two different reference groups: one with the bottom decile as the reference and another using the middle 5th/6th deciles as the reference.

#### 2.4.3 Model Performance and Calibration

We assessed the predictive performance of our models by evaluating discrimination with the Area Under the Receiver Operating Characteristic Curve (AUROC) and calibration with the Brier score (Pencina and D'Agostino 2015; Steyerberg et al. 2010) following the approach described in our previous study (Keaton et al. 2024). AUROC quantifies how well the model distinguishes between cases and controls, while the Brier score measures the accuracy of predicted probabilities (Pencina and D'Agostino 2015; Steyerberg et al. 2010). To determine the optimal risk threshold, we used the Youden Index, which identifies the point on the AUROC curve that maximizes the sum of sensitivity and specificity (Keaton et al. 2024; Schisterman et al. 2008).

We followed the STREGA guidelines (Little et al. 2009) for the reporting of this study (Table S1).

## 3 Results

### 3.1 Comparison of Results

#### 3.1.1 Clumps

The comparison of the number of clumps across all models is presented in Table 1. For example, for the SBP‐DBP analyses, we identified 1467 independent clumps from GenomicSEM(SBP‐DBP), whereas MTAG only yielded 1385 clumps for SBP and 1357 clumps for DBP. In fact, the single‐trait BP‐GWAS, analyzed on the same set of SNPS shared in common, obtained the most significant clumps: 1799 for SBP and 1743 for DBP (Table 1).

**TABLE 1:** Total number of clumps observed in univariate GWAS versus multivariate MTAG and GenomicSEM.

	SBP	DBP	PP	
**GWAS**	1799	1743	1530	
	**MTAG**			**GenomicSEM**
SBP‐DBP	1385	1357	—	1467
SBP‐PP	1378	—	1310	1395
DBP‐PP	—	1350	1305	1583

Abbreviations: DBP, Diastolic blood pressure; GWAS, Genome‐wide association study; GenomicSEM, genomic structural equation modeling; MTAG, multi‐trait analysis of GWAS; PP, pulse pressure; SBP, Systolic blood pressure.

#### 3.1.2 Comments on Beta/*p*‐Value Comparison Plots

We compared the *p*‐value results between multi‐trait GWAS and single‐trait GWAS. MTAG demonstrated nearly identical performance compared to its corresponding BP trait from the single‐trait GWAS, while GenomicSEM latent factors exhibited similar correlations with the two traits from which they were derived. We illustrate this pattern between multi‐trait and single‐trait GWAS using the SBP‐DBP model as an example in Figure 1. The correlations between beta values in GenomicSEM and the single‐trait GWAS were 0.930 for both SBP and DBP. Beta correlations for MTAG and the single‐trait GWAS were nearly identical (*r* = 0.999 for SBP and *r* = 0.998 for DBP). The comparisons across the two other 2‐trait models showed similar patterns (Supplementary Figure 1).

**FIGURE 1:**
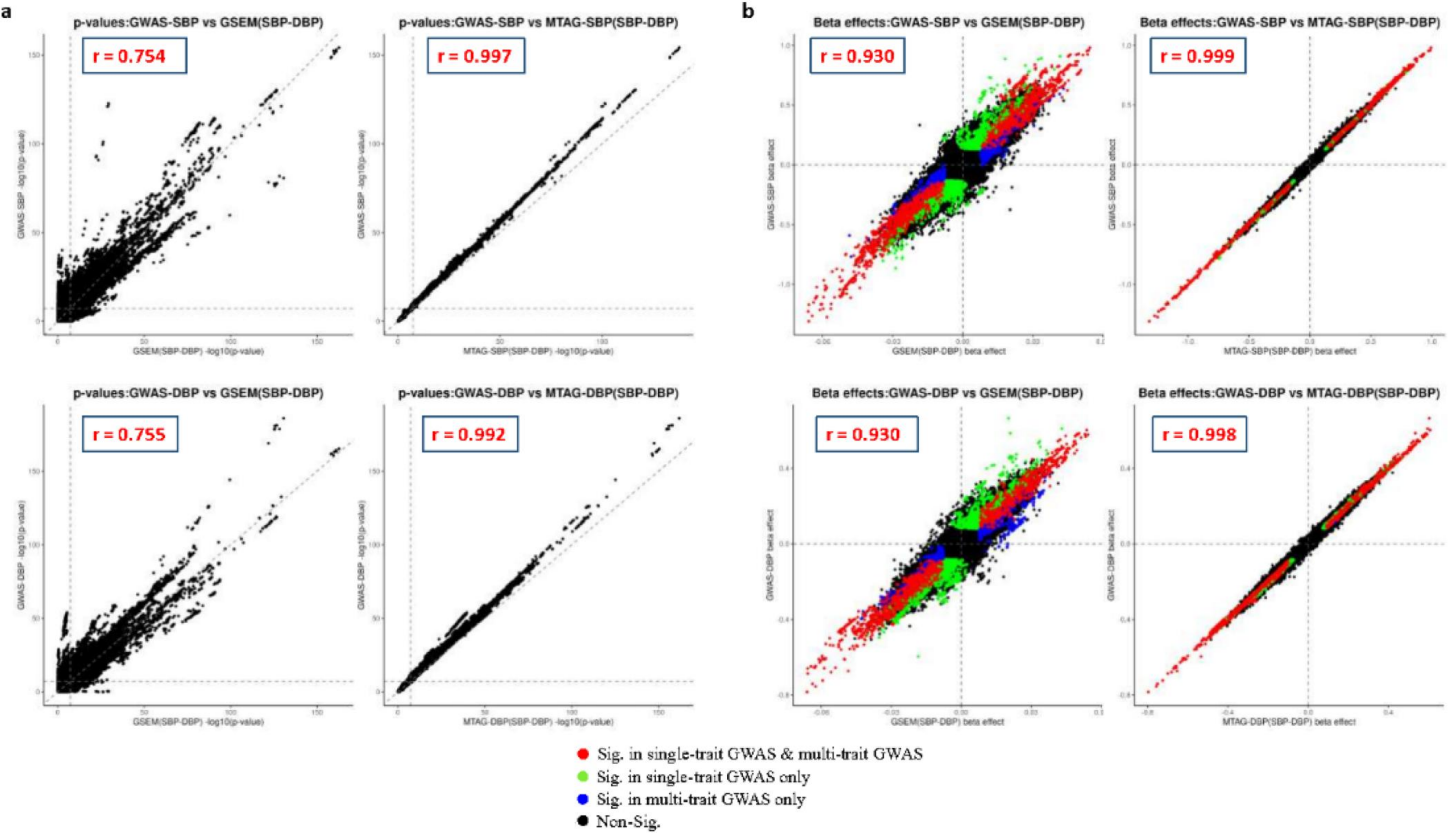
Comparison of (a) *p*‐values and (b) beta coefficients between multi‐trait and single‐trait GWAS for the SBP‐DBP two‐trait model. Single‐trait GWAS results are presented in rows (GWAS‐SBP and GWAS‐DBP), while multi‐trait GWAS results are shown in columns: GSEM (SBP‐DBP) and MTAG (SBP‐DBP). The Pearson Correlation Coefficient, *r*, is provided. DBP, diastolic blood pressure; GWAS, genome‐wide association study; SBP, systolic blood pressure; GSEM, genomic structural equation modeling; MTAG, multi‐trait analysis of GWAS.

It can be seen, however, from the *p*‐value comparison plots, that some multi‐trait GWAS analyses identified additional significant variants that were not genome‐wide‐significant in single‐trait GWAS. This motivates the investigation in Part (2), for searching for any novel loci from multi‐trait GWAS that were previously undetected in single‐trait GWAS, as we need to determine whether these additional significant variants are located in previously known or novel loci.

#### 3.1.3 Genetic Correlation Comparisons

We estimated the genetic correlations between each multi‐trait GWAS and single‐trait GWAS of BP traits (Table 2). MTAG exhibited nearly identical genetic correlations (*r*_g_ ≥ 0.999) with the corresponding single‐trait GWAS, regardless of the specific 2‐trait models used in their derivation. GenomicSEM latent factors generally showed consistent genetic correlations with their contributing traits. A notable exception was observed for the GenomicSEM(DBP‐PP). While its genetic correlations with GWAS DBP (*r*_g_ = 0.805) and GWAS PP (*r*_g_ = 0.813) were similar, it demonstrated a significantly stronger correlation with GWAS SBP (*r*_g_ = 0.998).

**TABLE 2:** Heritability and genetic correlation of blood pressure traits across GWAS, GenomicSEM, and MTAG.

Source	Trait	Heritability	GWAS			GenomicSEM			MTAG					
		(se)	SBP	DBP	PP	SBP‐DBP	SBP‐PP	DBP‐PP	SBP _(SBP‐DBP)_	SBP _(SBP‐PP)_	DBP _(SBP‐DBP)_	DBP_(DBP‐PP)_	PP _(SBP‐PP)_	PP _(DBP‐PP)_
GWAS	SBP	0.1280 (0.0050)	**1.000**											
	DBP	0.1179 (0.0048)	0.776	**1.000**										
	PP	0.1150 (0.0045)	0.840	0.310	**1.000**									
GenomicSEM	SBP‐DBP	0.1120 (0.0046)	0.943	0.941	0.613	**1.000**								
	SBP‐PP	0.1126 (0.0042)	0.959	0.566	0.959	0.812	**1.000**							
	DBP‐PP	0.1208 (0.0047)	0.998	0.805	0.813	0.958	0.945	**1.000**						
MTAG	SBP _(SBP‐DBP)_	0.1120 (0.0044)	1.000	0.786	0.830	0.949	0.954	0.999	**1.000**					
	SBP _(SBP‐PP)_	0.1119 (0.0043)	1.000	0.763	0.850	0.937	0.965	0.997	0.999	**1.000**				
	DBP _(SBP‐DBP)_	0.1044 (0.0042)	0.804	0.999	0.354	0.956	0.604	0.832	0.815	0.792	**1.000**			
	DBP _(DBP‐PP)_	0.1045 (0.0043)	0.805	0.999	0.355	0.956	0.605	0.833	0.816	0.793	1.000	**1.000**		
	PP _(SBP‐PP)_	0.1067 (0.0042)	0.864	0.354	0.999	0.649	0.971	0.840	0.855	0.874	0.398	0.399	**1.000**	
	PP _(DBP‐PP)_	0.1067 (0.0042)	0.864	0.354	0.999	0.649	0.971	0.840	0.855	0.874	0.398	0.399	1.000	**1.000**

*Note:* Subscripts in MTAG‐derived traits indicate the specific pair of traits on which the two‐trait MTAG model is based. Off‐diagonal entries represent genetic correlations between traits.

Abbreviations: DBP, diastolic blood pressure; GWAS, genome‐wide association study; GenomicSEM, genomic structural equation modeling; MTAG, multi‐trait analysis of GWAS; PP, pulse pressure; se, standard error; SBP, systolic blood pressure.

### 3.2 Novel Loci Discovery

We identified one single novel locus from multi‐trait GWAS: SNP rs12828318 on chr 12 (133,766,122 bp), which reached genome‐wide significance from the GenomicSEM(DBP‐PP) analysis (*p* = 4.93 × 10^−8^), but not in any of the original single‐trait GWAS (*p* = 2.09 × 10^−7^ for SB*P*; *p* = 4.29 × 10^−7^ for DB*P*; *p* = 2.42 × 10^−4^ for PP). According to the DBP‐GWAS, the minor allele G (effect allele frequency = 0.18) decreases DBP by 0.105 mmHg. The nearest gene is *ZNF268 (Zinc Finger Protein 268)* (Supplementary Figure 2). This novel SNP rs12828318 is > 500kb away from, and not in LD (*r* ^2^ < 0.1) with any previously published BP SNPs. GWAS catalog links the *ZNF268* gene to waist‐hip ratio, type 2 diabetes, cardiovascular disease (CVD), coronary artery disease (CAD), smoking, osteoarthritis, and educational attainment (Table S2).

Functional annotation using SNPannotator v0.2.6.0 (Ani et al. 2023) showed that rs12828318 is intronic and associated with waist‐hip ratio (Table S3). Three linked SNPs (LD *r* ^2^ > 0.8) were associated with CVD (rs36174733), CAD (rs36127550), and brain‐related traits (rs11147235). Using the Open Targets Genetics Platform (https://genetics.opentargets.org/), which prioritizes potential causal variants at trait‐associated loci (Mountjoy et al. 2021), we performed lookups of gene expression and phenome‐wide associations (PheWAS), which showed that rs12828318 was associated with expression levels of nine genes, with *ZNF268* being the nearest coding gene (8127 bp from the canonical TSS) (Table S4), and PheWAS lookups revealed an association with hypertension (*β* = −0.00661, *p* = 2.1 × 10^−9^).

### 3.3 Heritability

We did not observe improvements in heritability in the multi‐trait GWAS, with all heritability estimates from MTAG and GenomicSEM being similar to those from the single‐trait GWAS (Table 2). The heritability estimates derived from MTAG were highly consistent for the same trait, with SBP showing the highest estimate (*h* ^2^ = 0.112), followed by PP (*h* ^2^ = 0.107) and DBP (*h* ^2^ = 0.104). Among all GenomicSEM latent factors, the DBP‐PP factor exhibited the highest heritability (*h* ^2^ = 0.121).

### 3.4 Polygenic Risk Scores and Hypertension Risk Prediction

#### 3.4.1 PRS and Variance Explained

We generated five different PRS for the two‐trait SBP‐DBP model using SBayesRC. Pearson correlation between the five PRS exhibited a pattern consistent with the genetic correlations among their corresponding traits (Table 3).

**TABLE 3:** Pearson correlation of SBayesRC PRSs derived from GWAS, GenomicSEM, and MTAG in the SBP–DBP two‐trait model.

Source	Trait	GWAS		GenomicSEM	MTAG	
		SBP	DBP	SBP‐DBP	SBP _(SBP‐DBP)_	DBP _(SBP‐DBP)_
GWAS	SBP	**1.000**				
	DBP	0.740	**1.000**			
GenomicSEM	SBP‐DBP	0.917	0.915	**1.000**		
MTAG	SBP _(SBP‐DBP)_	0.980	0.758	0.922	**1.000**	
	DBP _(SBP‐DBP)_	0.762	0.987	0.937	0.776	**1.000**

Abbreviations: DBP, diastolic blood pressure; GWAS, genome‐wide association study; GenomicSEM, genomic structural equation modeling; MTAG, multi‐trait analysis of GWAS; PP, pulse pressure; SBP, systolic blood pressure.

When we evaluated the variance explained by SBayesRC PRS in SBP, DBP, and hypertension, we found that PRS derived from MTAG and GenomicSEM explained a similar amount of variance as those from the single‐trait GWAS. For example, for SBP, the variance explained by PRS was 11.37%, 11.41%, and 10.98% using PRS_GWAS‐SBP_, PRS_MTAG‐SBP_, and PRS_GenomicSEM_, respectively, and similarly for DBP. For hypertension, the highest variance explained was observed in models including SBP and DBP, with values of 7.31% for GWAS, 7.19% for MTAG, and 6.97% for GenomicSEM. The other PRSs derived from GWAS and MTAG also showed similar results (Table 4).

**TABLE 4:** Variance explained and incremental *R* ^2^ by SBayesRC PRSs across GWAS, MTAG, and GenomicSEM for SBP, DBP, and hypertension in the Lifelines dataset (*n* = 10,210, European ancestry).

SBP	VE	Δ*R* ^2^
Model 1	0.003	—
Model 1+PRS(GWAS‐SBP)	0.117	0.1137
Model 1+PRS(MTAG‐SBP)	0.117	0.1141
Model 1+PRS(GenomicSEM)	0.113	0.1098
**DBP**	**VE**	**Δ*R*** ^**2**^
Model 1	0.000	—
Model 1+PRS(GWAS‐SBP)	0.119	0.1193
Model 1+PRS(MTAG‐SBP)	0.120	0.1196
Model 1+PRS(GenomicSEM)	0.108	0.1084
**HTN**	**VE**	**Δ*R*** ^**2**^
Model 1	0.287	—
Model 1+PRS(GWAS‐SBP)+PRS(GWAS‐DBP)	0.360	0.0731
Model 1+PRS(MTAG‐SBP)+PRS(MTAG‐DBP)	0.359	0.0719
Model 1+PRS(GWAS‐SBP)	0.357	0.0692
Model 1+PRS(GWAS‐DBP)	0.343	0.0554
Model 1+PRS(MTAG‐SBP)	0.356	0.0683
Model 1+PRS(MTAG‐DBP)	0.345	0.0574
Model 1+PRS(GenomicSEM)	0.357	0.0697

*Note:* Model 1 in SBP/DBP: SBP/DBP residual~PC1+PC2+PC3+PC4+PC5+PC6+PC7+PC8+PC9+PC10

Model 1 in HTN: HTN~age+age2+sex+BMI+PC1+PC2+PC3+PC4+PC5+PC6+PC7+PC8+PC9+PC10

Abbreviations: DBP, diastolic blood pressure; GWAS, genome‐wide association study; GenomicSEM, genomic structural equation modeling; HTN, hypertension; MTAG, multi‐trait analysis of GWAS; PRS, polygenic risk score; PCs, principal components; SBP, systolic blood pressure; Δ*R* ^2^, incremental variance explained; VE, variance explained.

#### 3.4.2 Association of PRS and Hypertension

We tested the association between the deciles of the PRS distribution and hypertension using different PRS approaches. In general, the results from GenomicSEM were slightly lower than those from the single‐trait GWAS and MTAG in both the average and joint models. For example, when comparing the top decile with the bottom decile in the fully adjusted model, the OR for the GenomicSEM‐derived PRS was 10.14 (95% CI: 7.79, 13.28). In contrast, the OR for the average GWAS and MTAG models were 10.76 (95% CI: 8.22, 14.18) and 11.23 (95% CI: 8.59, 14.80), respectively. Furthermore, in the joint model, the OR for GWAS was 13.69 (95% CI: 9.96, 18.83), and for MTAG it was 12.93 (95% CI: 9.44, 17.72). The results were similar when the reference category was the 5th and 6th deciles (Table S5).

#### 3.4.3 Model Performance

The area under the receiver operating characteristic curve (AUROC) for Model 1, containing only covariates, was 0.791 (95% CI: 0.781, 0.801) (Table 5). The AUROC increased to 0.826 (95% CI: 0.817, 0.836) when adding the single‐trait GWAS PRS (both PRS for SBP and DBP) and remained the same at 0.826 (95% CI: 0.816, 0.835) when instead the MTAG PRS (both PRS for SBP and DBP) was added to Model 1. Similarly, the AUROC for the model with GenomicSEM PRS was 0.825 (95% CI: 0.815, 0.834). The Brier scores for Models 1 (0.142), 2 (0.130), 3 (0.130), and 4 (0.131) suggest good calibration across all models. Similarly, the Youden index showed an increase in all models compared to the first, with values of 1.428 for Model 1, 1.504 for Model 2, 1.504 for Model 3, and 1.502 for Model 4.

**TABLE 5:** Performance and calibration statistics of hypertension model incorporating SBayesRC PRSs derived from GWAS, MTAG, and GenomicSEM in Lifelines.

	Model 1	Model 2	Model 3	Model 4
	(only covariates)	(covariates+GWAS PRS)	(covariates+MTAG PRS)	(covariates+ GenomicSEM PRS)
AUROC (95% CI)	0.791 (0.781–0.801)	0.826 (0.817–0.836)	0.826 (0.816–0.835)	0.825 (0.815–0.834)
Brier Score	0.142	0.130	0.130	0.131
Youden cut point				
Specificity	0.70	0.74	0.72	0.76
Sensitivity	0.73	0.76	0.78	0.75
Youden index	1.428	1.504	1.504	1.502
NPV	0.893	0.910	0.914	0.906
PPV	0.428	0.476	0.467	0.486
Percentile of the total sample at the cut point	57.8%	62.1%	60.5%	63.8%
NRI	—	0.080 (0.063–0.096)	0.081 (0.065–0.098)	0.080 (0.063–0.097)

Abbreviations: AUROC, area under the receiver operating characteristic curve; CI, confidence interval; GenomicSEM, genomic structural equation modeling; GWAS, genome‐wide association study; MTAG, multi‐trait analysis of GWAS; NRI, net reclassification improvement; NPV, negative predictive value; PPV, positive predictive value; PRS, polygenic risk score; PCs, principal components.

*Note:* Model 1: HTN~age+age2+sex+BMI+PC1+PC2+PC3+PC4+PC5+PC6+PC7+PC8+PC9+PC10.

Model 2: Model 1+PRS(GWAS‐SBP)+PRS(GWAS‐DBP).

Model 3: Model 1+PRS(MTAG‐SBP)+PRS(MTAG‐DBP).

Model 4: Model 1+PRS(GenomicSEM).

SBP and DBP in MTAG were derived from SBP‐DBP two‐trait model.

## 4 Discussion

In this study, we applied multivariate approaches using GenomicSEM and MTAG to analyze systolic BP, diastolic BP, and pulse pressure, based on the latest GWAS data from over one million individuals of European ancestry. We compared the results from all bivariate multi‐trait analyses with single‐trait GWAS in terms of loci discovery, SNP‐based heritability estimates, and the association between PRS and hypertension. However, in the current scenario, characterized by complete sample overlap across BP GWAS, we found limited gains from multi‐trait BP‐GWAS, with only one novel locus being identified, and no improvement in heritability estimates or PRS performance compared to single‐trait GWAS. These findings quantify the limited benefit of multi‐trait methods for BP‐GWAS with complete sample overlap.

Our novel locus, *ZNF268*, is a gene with multiple splice isoforms expressed in fetal tissues and tumor cell lines (Shao et al. 2006). It plays a role in fetal development, particularly in the liver and blood cells, and may act as a transcriptional regulator involved in cellular differentiation and pathogenesis (Shao et al. 2006; Sun et al. 2004). Previous GWASs have associated this locus with waist‐to‐hip ratio (Lotta et al. 2018), CVD (Kichaev et al. 2019), CAD (Tcheandjieu et al. 2022), and type 2 diabetes (Vujkovic et al. 2020), but its precise role in BP regulation remains unclear.

We believe the most likely reason for there being limited gains in results from our multi‐trait BP‐GWAS vs single‐trait results, is because the SBP‐GWAS, DBP‐GWAS, and PP‐GWAS all originated from exactly the same meta‐analysis dataset, with no distinct samples across traits, that is, in this setting of complete sample overlap. In contrast, the original study using MTAG demonstrated the benefit of meta‐analyzing data from multiple traits, where the GWAS of these different traits had been conducted on different datasets with some non‐overlapping samples (Turley et al. 2018). This is advantageous in the study of some diseases, where phenotypes are not as routinely collected as BP, for example, and may not always be available, or may be defined differently with slightly different traits within different cohorts, limiting the sample sizes of single‐trait GWAS meta‐analyses when like‐with‐like data cannot be so easily combined. Instead, of course, BP data, in reality, will always also include both DBP and PP on any individual with an available SBP measurement, because as soon as someone's BP is measured, both SBP and DBP are generated, and PP values can be directly derived. Therefore, any multi‐trait analysis of BP traits will usually have complete sample overlap. GenomicSEM and MTAG are both expected to yield increases in statistical power relative to univariate GWASs when the constituent univariate GWASs exhibit lower levels of sample overlap (Grotzinger et al. 2019; Turley et al. 2018). By implementing an advanced extension of LDSC, both methods are robust to sample overlap (Bulik‐Sullivan et al. 2015). However, under conditions of complete sample overlap, the limited gain in power, coupled with these rigorous corrections, resulted in less significant multi‐trait association findings.

One other reason for the limited gain of our multi‐trait BP analyses may be because the single‐BP GWAS results are already so highly powered, with *N* > 1 million, and over 2100 independent BP signals (Keaton et al. 2024). Multi‐trait GWAS may be more beneficial for other traits, with smaller single‐trait GWAS sample sizes, with more power still remaining. Notably, all four loci previously uncovered in the smaller 2015 COGENT BP study (Zhu et al. 2015) with a multivariate approach have now reached significance in the most recent single‐trait BP‐GWAS analysis due to increased sample size.

This study has several strengths. First, the large sample size provided robust statistical power. Second, the comparison between multivariate and univariate approaches allowed a systematic evaluation of their respective utility. Third, the identification of a novel locus was conducted using a validated analytical pipeline consistent with previous studies (Keaton et al. 2024), ensuring methodological reliability. Moreover, external validation in an independent cohort (Lifelines) enhanced the real‐world applicability of the PRSs. Finally, the use of a Bayesian‐based PRS method (SBayesRC) incorporating functional annotations contributed to improved prediction accuracy.

However, the study also has some limitations. Firstly, rare genetic variation is not covered within the GWAS data (MAF > 1%) used in this study. Therefore, alternative approaches are still needed for novel genetic discovery of low‐frequency variants.

An important limitation is the exclusive use of European ancestry data, as the original single‐trait GWAS was European ancestry only, which limits the generalizability of findings to more diverse populations. We note that the multi‐trait COGENT study (Zhu et al. 2015) from 2015 was an African ancestry dataset. It is well recognized now that improvements in GWAS discovery can be achieved from multi‐ancestry analyses, and the GWAS field is striving to improve ancestry diversity. This provides a particular challenge for statistical geneticists for the future with respect to alternative GWAS methodologies, because multi‐trait approaches that we investigate here cannot yet take multi‐ancestry heterogeneity into account. In particular, both methods here, Genomic‐SEM and MTAG, require the use of LDSC, which becomes challenging in analyses of multi‐ancestry data, if different LD reference panels are required from different ancestries. The implementation of multi‐trait GWAS approaches within multi‐ancestry datasets still requires further methodological advance (Levin et al. 2022).

Finally, the absence of a three‐trait model integrating SBP, DBP, and PP ‐due to collinearity‐ restricted the evaluation of more complex BP relations. The strong linear relationships among SBP, DBP, and PP, with PP calculated as a linear combination of SBP and DBP, constrained us to two‐trait models, while their moderate‐to‐high genetic correlations left limited scope for the identification of novel loci or improvement of PRS prediction. Incorporating a broader range of related phenotypes may help overcome this limitation. Previous studies have shown that incorporating information from multiple traits significantly optimizes the accuracy of genetic risk prediction (Norland et al. 2024; Sinnott‐Armstrong et al. 2021). Based on our results, we recommend for the future that the flexibility of multi‐trait GWAS may be better leveraged when integrating novel, independent GWAS data together on a variety of other BP‐related traits, rather than only performing multi‐trait analyses of BP traits alone.

However, while integrating additional related traits is encouraged, recent studies in the literature have shown that a linear weighted combination of single‐trait PRS may be a more straightforward and effective approach for improving PRS prediction, compared to constructing multi‐trait GWAS‐derived PRS. For example, a linear weighted combination of PRS for a broad range of traits has improved the prediction of coronary heart disease (Norland et al. 2024).

Furthermore, although SBP and DBP are positively correlated, a genetic variant may exert discordant effects on these traits (Zhu et al. 2015). Neither MTAG nor GenomicSEM capture mixed effect directions (Hindley et al. 2023). In our study, 18 independent lead SNPs reached genome‐wide significance in GWAS‐SBP and GWAS‐DBP but exhibited opposite directions of effect (Supplementary Table 6). This antagonistic pleiotropy rendered them particularly significant in GWAS‐PP. However, these variants did not confer any advantage in multi‐trait analyses: they were not significant and exhibited negligible effect sizes in Genomic SEM and showed attenuated significance relative to single‐trait GWAS in MTAG. These findings may partly account for the fewer loci identified by multi‐trait GWAS compared to single‐trait approaches. Bivariate causal mixture model (MixeR) (Frei et al. 2019) and Local Analysis of Variant Association (Werme et al. 2022) can elucidate the biological underpinnings of concordant and discordant effects at the variant or regional level. Future studies may benefit from integrating this information on a genome‐wide scale to enhance locus discovery and refine PRS prediction.

In conclusion, multi‐trait methods have previously demonstrated power advantages over conventional GWAS, often claiming greater discoveries across a range of traits (Turley et al. 2018). However, in the context of BP traits analyzed under complete sample overlap, our findings indicate that multivariate approaches offer minimal added benefit over single‐trait GWAS. Notably, the results from our study should not be interpreted as providing a general assessment of the performance of multi‐trait methods across all complex traits, or across all study design scenarios. Any conclusions are trait‐specific, and it is possible that multi‐trait GWAS approaches may be more beneficial for other common complex traits. Many of the multi‐trait studies in the literature with success stories, including the original MTAG study (Turley et al. 2018), have been investigating psychiatric traits. Compared to BP‐GWAS, the sample sizes of GWAS of psychiatric traits are smaller. Furthermore, psychiatric traits are more heterogeneous phenotypically, where outcomes within a GWAS meta‐analysis may be measured according to slightly different trait definitions and derivations across the contributing studies, leading to a study design which is less likely to have complete sample overlap, like our current BP‐GWAS investigation.

As recommendations for future BP‐PRS analyses, if using PRS for prediction models, we would suggest simply performing a multivariate model of the single‐trait SBP‐PRS and DBP‐PRS together, as this was not outperformed by our more complex multi‐trait GWAS‐derived PRS. However, if using BP‐PRS in interaction model analyses, for example, analysts may still find some practical benefits to having only one overall BP‐PRS from GenomicSEM.

As recommendations for study design of future BP‐GWAS, we expect that future novel BP genetic discovery is more likely to come from the addition of new data with increased sample sizes across multiple diverse ancestries, and from analyses considering rare variants as well as common variants.

## UMCG Genetics Lifelines Initiative (UGLI) Group Author: Lifelines Cohort Study

Raul Aguirre‐Gamboa (1), Patrick Deelen (1), Lude Franke (1), Jan A Kuivenhoven (2), Esteban A Lopera Maya (1), Ilja M Nolte (3), Serena Sanna (1), Harold Snieder (3), Morris A Swertz (1), Peter M. Visscher (3,4), Judith M Vonk (3), Cisca Wijmenga (1), Naomi Wray (4)

1. *Department of Genetics, University of Groningen, University Medical Center Groningen, The Netherlands*.
2. *Department of Pediatrics, University of Groningen, University Medical Center Groningen, The Netherlands*.
3. *Department of Epidemiology, University of Groningen, University Medical Center Groningen, The Netherlands*.
4. *Institute for Molecular Bioscience, The University of Queensland, Brisbane, Queensland, Australia*.

## Ethics Statement

The Lifelines protocol was approved by the UMCG Medical Ethics Committee under number 2007/152. All participants provided informed consent.

## Conflicts of Interest

The authors declare no conflicts of interest.

## Supporting information


**Figure S1:** Pairwise comparisons across GWAS, GSEM, and MTAG summary statistics (left: beta coefficients; right: P‐values; slides 1–9: SBP; slides 10–18: DBP; slides 19–27: PP). For the beta coefficient comparisons, point colours denote the significance status of SNPs across the respective summary statistics. For the P‐value comparisons, the horizontal and vertical dashed lines indicate the genome‐wide significance threshold (*P* = 5 × 10^−8^; –log_10_ *P* ≈ 7.3). Abbreviations: SBP, systolic blood pressure; DBP, diastolic blood pressure; GWAS, genome‐wide association study; MTAG, multi‐trait analysis of GWAS; GSEM, genomic structural equation modelling.
**Figure S2:** Locus Zoom plot of the novel locus (*ZNF268*) on chromosome 12 identified from the GSEM(DBP‐PP) analysis, centred ±500 kb around the sentinel SNP rs12828318.

**Table S1:** STREGA Statement—checklist of items that should be included in genetic association studies.
**Table S2**: In‐Silico Analysis of ZNF268: GWAS Catalog Search. SNP, single nucleotide polymorphism; GWAS, genome‐wide association study; OR, odds ratio; CI, confidence interval; CNV, copy number variation; PRS, polygenic risk score; EFO, Experimental Factor Ontology; MONDO, Monarch Disease Ontology; NR, not reported; CHR_POS, chromosomal position; NA, not available.
**Table S3**: In‐Silico Analysis of rs12828318: SNP Annotation Using SNP Annotator. SNP, single nucleotide polymorphism; Chr, chromosome; Pos, chromosomal coordinate; CADD, Combined Annotation Dependent Depletion; LD, pairwise linkage disequilibrium (*r* ^2^) between gSNP and linked SNP.
**Table S4**: Evidence summary linking rs12828318 to different genes. Results from Open Targets Genetics. V2G, Variant to Gene; TSS, transcription start site; eQTL; expression quantitative trait locus.
**Table S5**: Association of PRSs deciles hypertension. SBP and DBP in MTAG were derived from SBP‐DBP two‐trait model. SBP, systolic blood pressure; DBP, diastolic blood pressure; PRS, polygenic risk score; GWAS, genome‐wide association study; MTAG, multi‐trait analysis of GWAS; GSEM, genomic structural equation modelling; CI, confidence interval.
**Table S6**: Eighteen independent lead SNPs with significant discordant effects on SBP and DBP associations. SNP, single nucleotide polymorphism; CHR, chromosome; EAF, effect allele frequency; GWAS, genome‐wide association study; PP, pulse pressure; SBP, systolic blood pressure; DBP, diastolic blood pressure; MTAG, multi‐trait analysis of GWAS; GenomicSEM, genomic structural equation modeling.

## Data Availability

This study, as input data, used the BP‐GWAS summary statistics from Keaton et al, Nature Genetics 2024,2024, which are publicly available on GWAS catalog (https://www.ebi.ac.uk/gwas) with data accession codes GCST90310294, GCST90310295, and GCST90310296 for SBP, DBP, and PP, respectively.
