# Supplementary material for: Multi‐Trait GWAS Analyses of Blood Pressure Traits Provide Limited Gains in Locus Discovery or Polygenic Risk Score Performance Compared to Single‐Trait GWAS
Source: Genet Epidemiol. 2026 Oct 22;50(8):e70062. doi: 10.1002/gepi.70062 (PMC13688232; doi:10.1002/gepi.70062)
Supplement: Supplementary file 1 [file GEPI-50-0-s001.pdf]

Supplementary Figures

Sup Fig 1
(beta/P-value comparison plots)

Sup Fig 1
(SBP-DBP model)

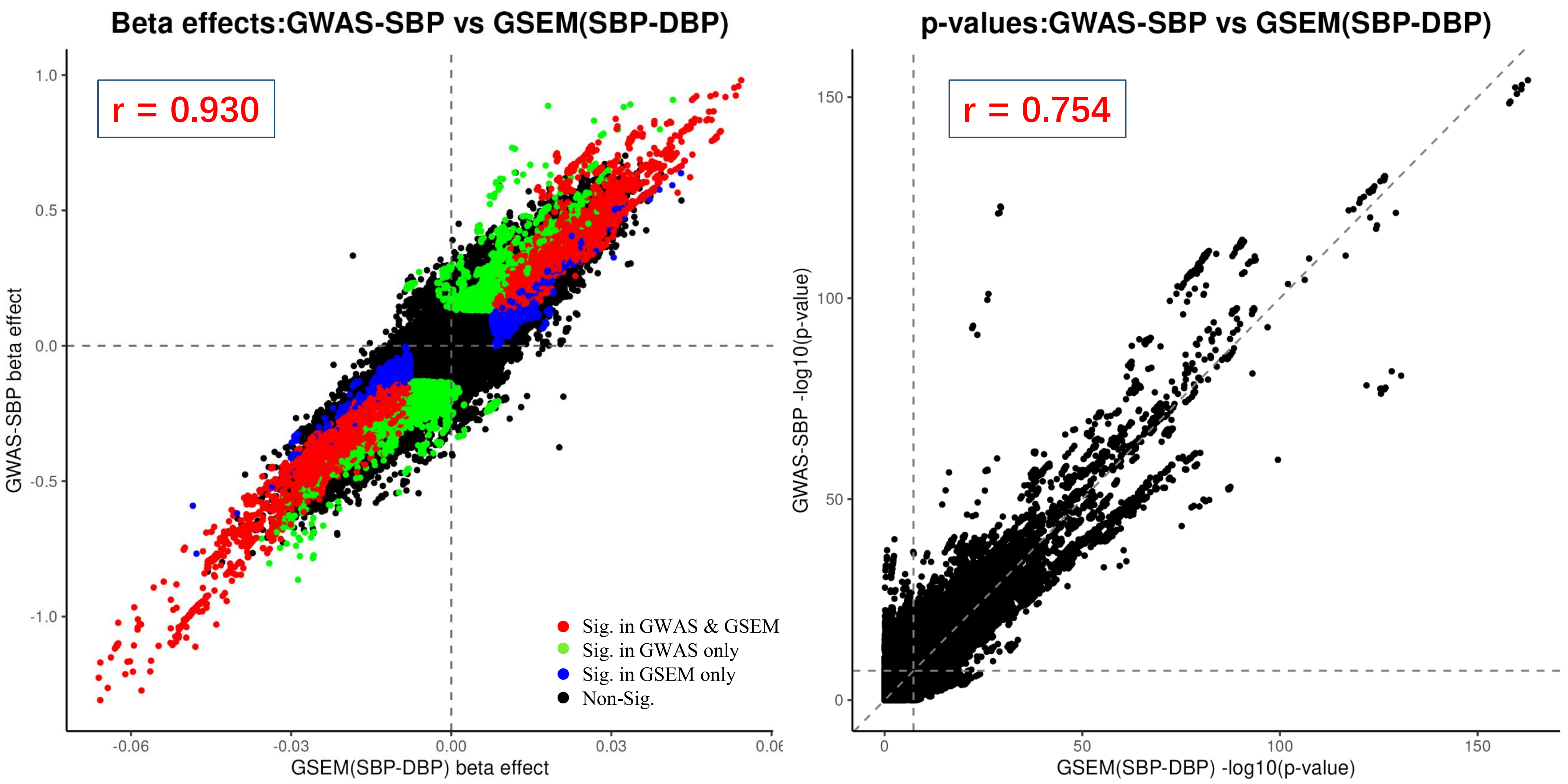
Beta effects:GWAS-SBP vs GSEM(SBP-DBP)
r = 0.930
GWAS-SBP beta effect
GSEM(SBP-DBP) beta effect
1.0
0.5
0.0
-0.5
-1.0
-0.06
-0.03
0.00
0.03
Sig. in GWAS & GSEM
Sig. in GWAS only
Sig. in GSEM only
Non-Sig.
p-values:GWAS-SBP vs GSEM(SBP-DBP)
r = 0.754
GWAS-SBP -log10(p-value)
GSEM(SBP-DBP) -log10(p-value)
150
100
50
0

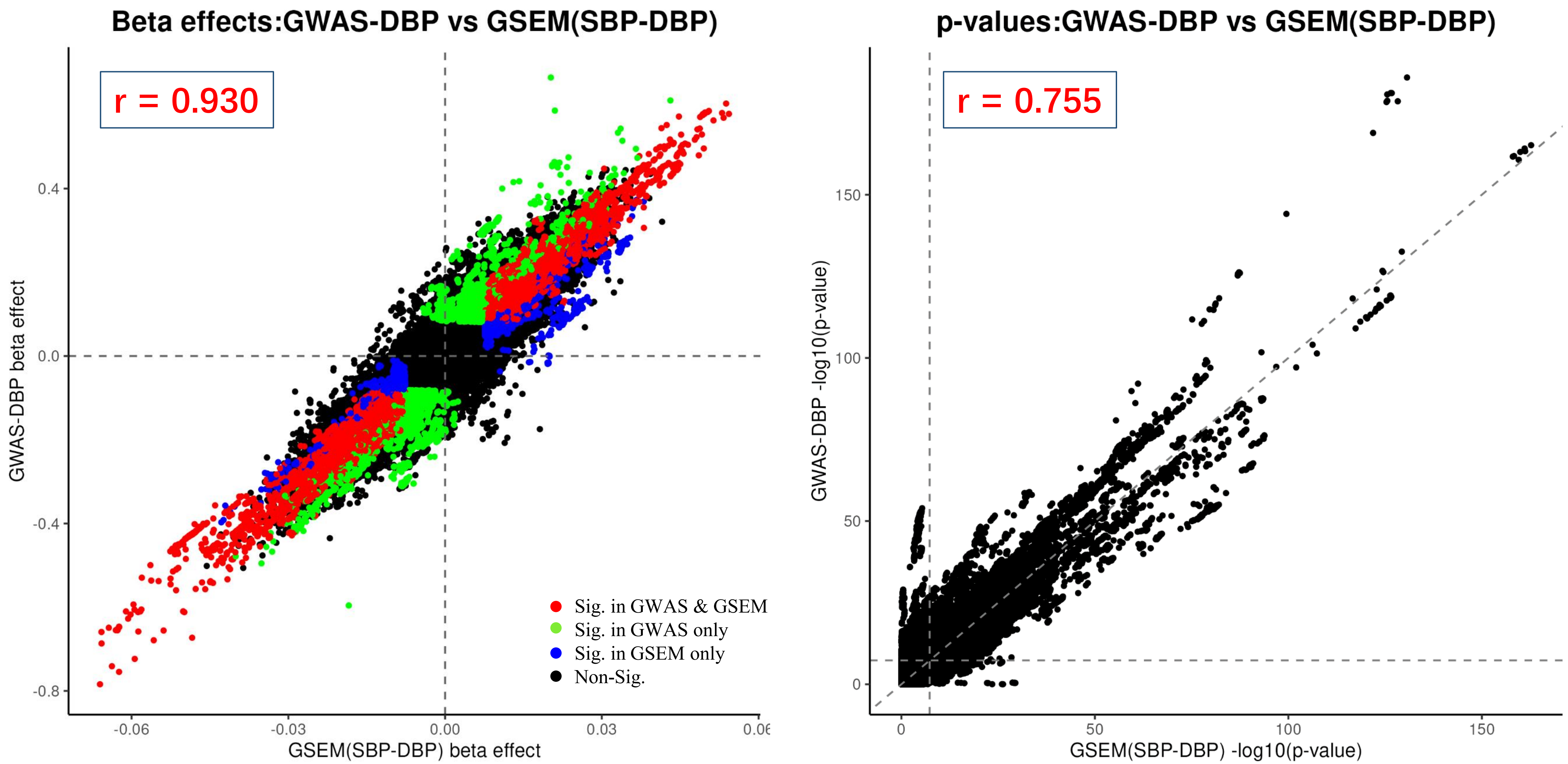
Beta effects:GWAS-DBP vs GSEM(SBP-DBP)
r = 0.930
GWAS-DBP beta effect
GSEM(SBP-DBP) beta effect
0.4
0.0
-0.4
-0.8
-0.06
-0.03
0.00
0.03
Sig. in GWAS & GSEM
Sig. in GWAS only
Sig. in GSEM only
Non-Sig.
p-values:GWAS-DBP vs GSEM(SBP-DBP)
r = 0.755
GWAS-DBP -log10(p-value)
GSEM(SBP-DBP) -log10(p-value)
150
100
50
0
0
50
100
150

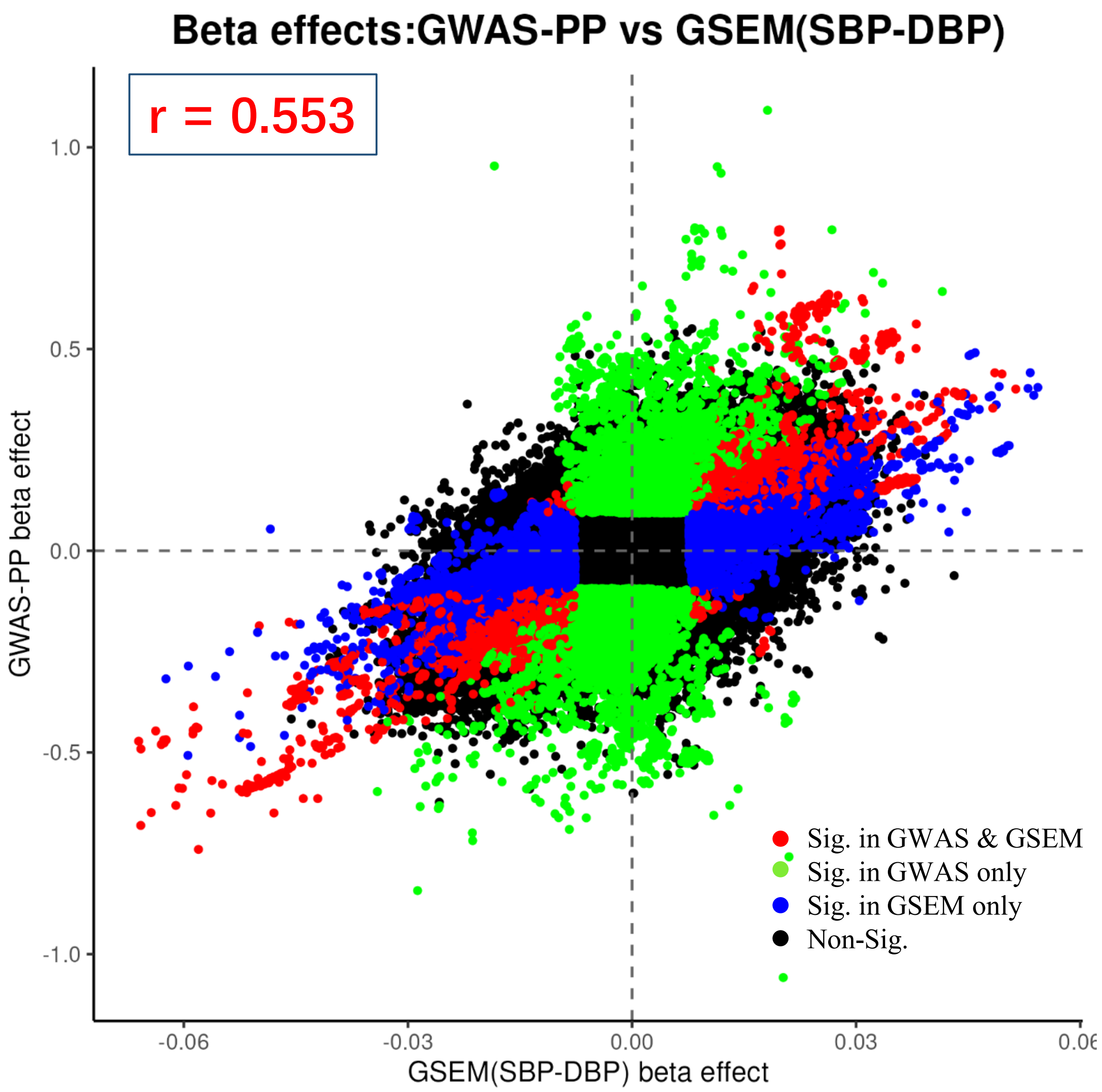
Beta effects:GWAS-PP vs GSEM(SBP-DBP)
r = 0.553
GWAS-PP beta effect
GSEM(SBP-DBP) beta effect
Sig. in GWAS & GSEM
Sig. in GWAS only
Sig. in GSEM only
Non-Sig.

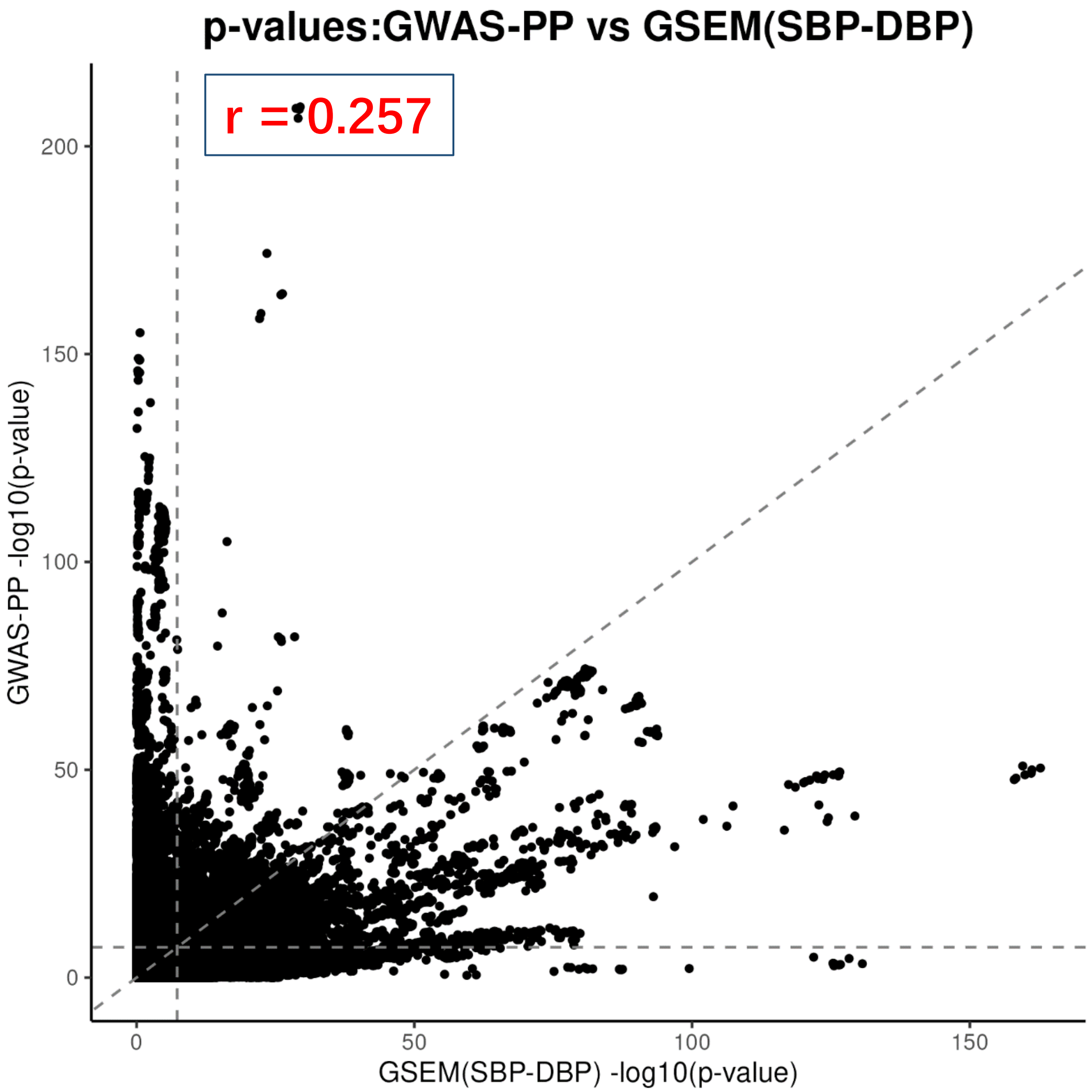
p-values:GWAS-PP vs GSEM(SBP-DBP)
r = 0.257
GWAS-PP -log10(p-value)
GSEM(SBP-DBP) -log10(p-value)

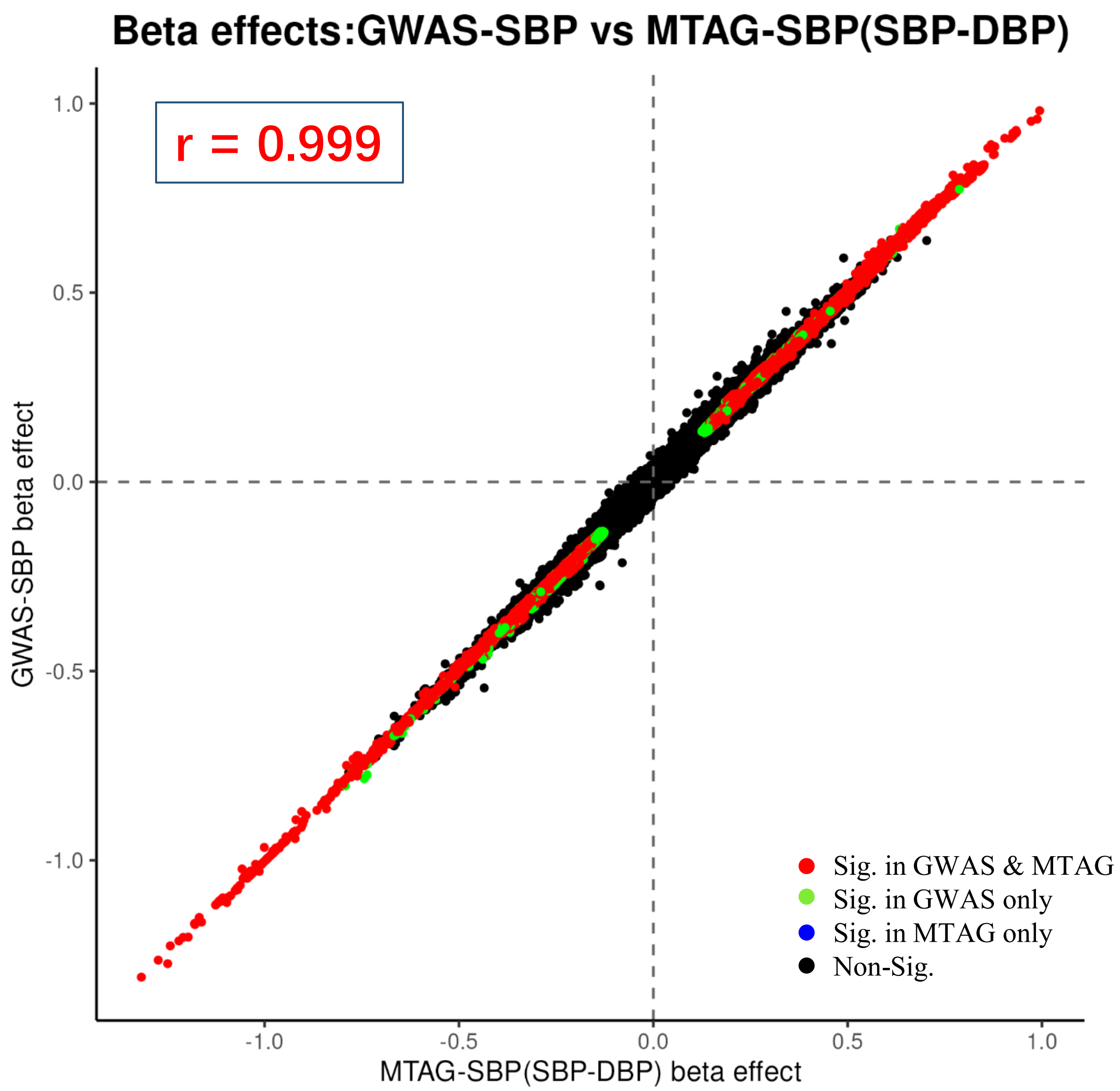
Beta effects:GWAS-SBP vs MTAG-SBP(SBP-DBP)
r = 0.999
GWAS-SBP beta effect
MTAG-SBP(SBP-DBP) beta effect
1.0
0.5
0.0
-0.5
-1.0
-1.0
-0.5
0.0
0.5
1.0
Sig. in GWAS & MTAG
Sig. in GWAS only
Sig. in MTAG only
Non-Sig.
p-values:GWAS-SBP vs MTAG-SBP(SBP-DBP)
r = 0.997
GWAS-SBP -log10(p-value)
MTAG-SBP(SBP-DBP) -log10(p-value)
150
100
50
0
0
50
100

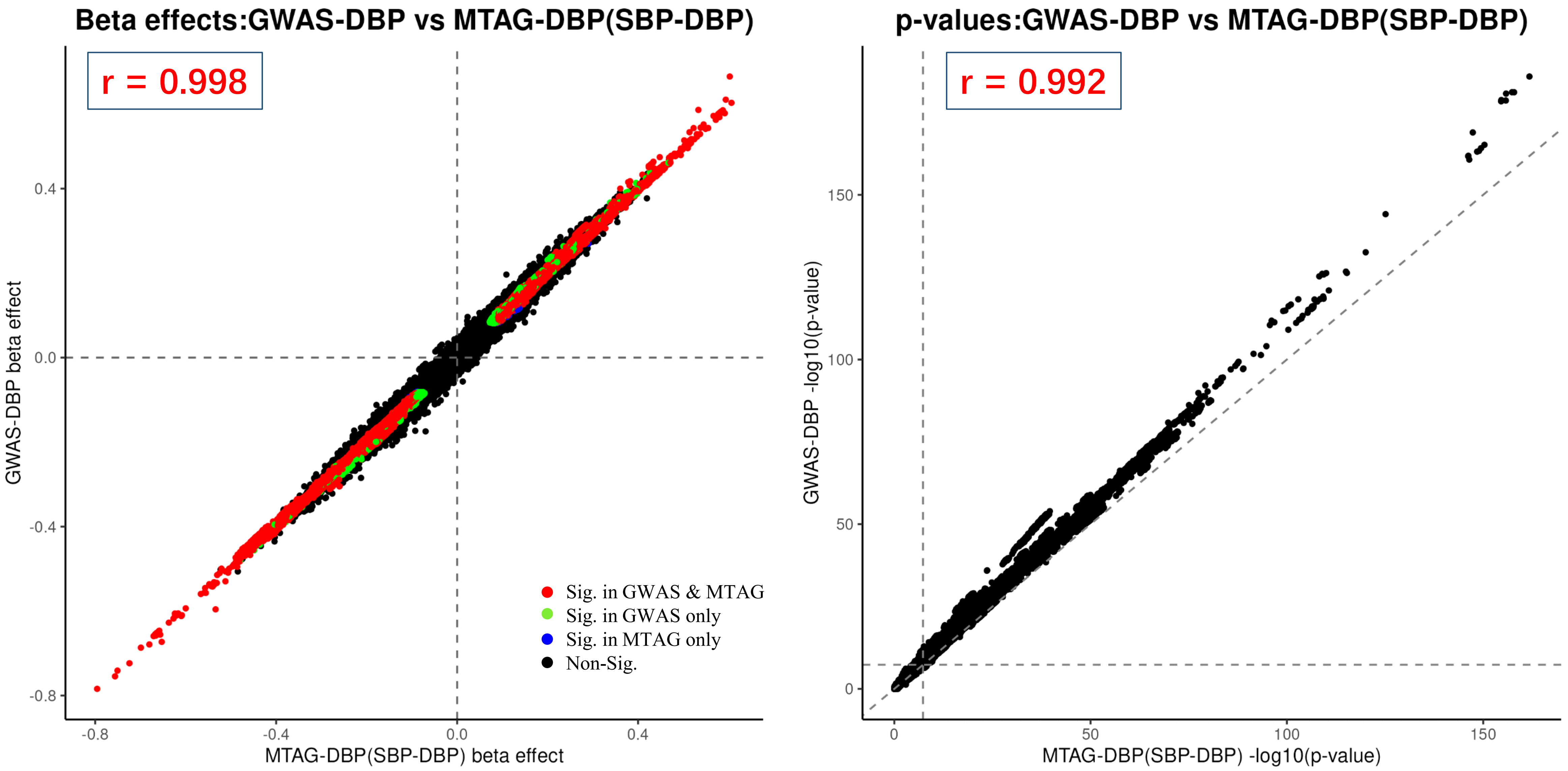
Beta effects:GWAS-DBP vs MTAG-DBP(SBP-DBP)
r = 0.998
GWAS-DBP beta effect
0.4
0.0
-0.4
-0.8
-0.8
-0.4
0.0
0.4
MTAG-DBP(SBP-DBP) beta effect
Sig. in GWAS & MTAG
Sig. in GWAS only
Sig. in MTAG only
Non-Sig.
p-values:GWAS-DBP vs MTAG-DBP(SBP-DBP)
r = 0.992
GWAS-DBP -log10(p-value)
150
100
50
0
0
50
100
150
MTAG-DBP(SBP-DBP) -log10(p-value)

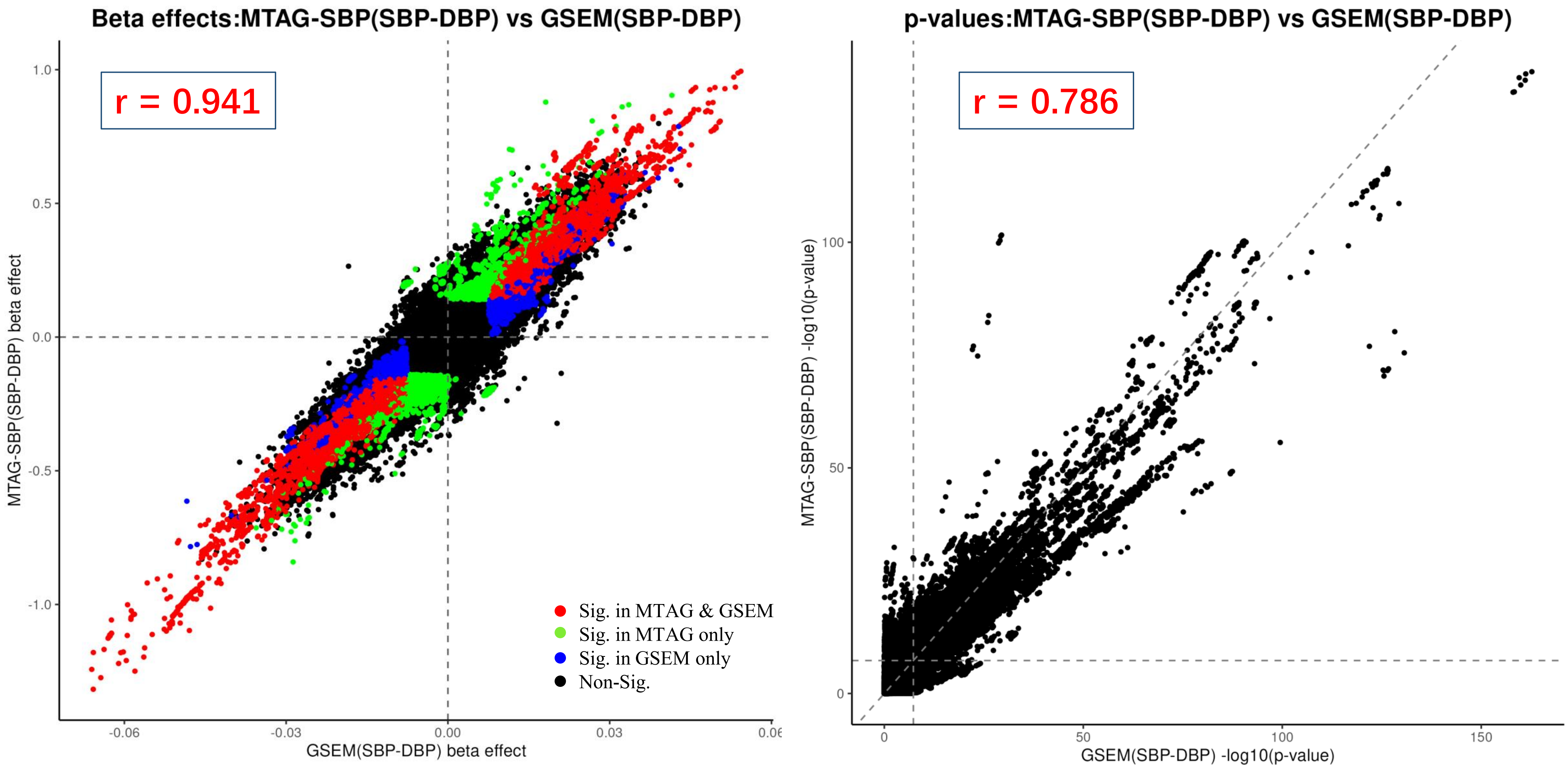

Beta effects:MTAG-SBP(SBP-DBP) vs GSEM(SBP-DBP)
r = 0.941
MTAG-SBP(SBP-DBP) beta effect
GSEM(SBP-DBP) beta effect
1.0
0.5
0.0
-0.5
-1.0
-0.06
-0.03
0.00
0.03
0.06
Sig. in MTAG & GSEM
Sig. in MTAG only
Sig. in GSEM only
Non-Sig.
p-values:MTAG-SBP(SBP-DBP) vs GSEM(SBP-DBP)
r = 0.786
MTAG-SBP(SBP-DBP) -log10(p-value)
GSEM(SBP-DBP) -log10(p-value)
100
50
0
0
50
100
150

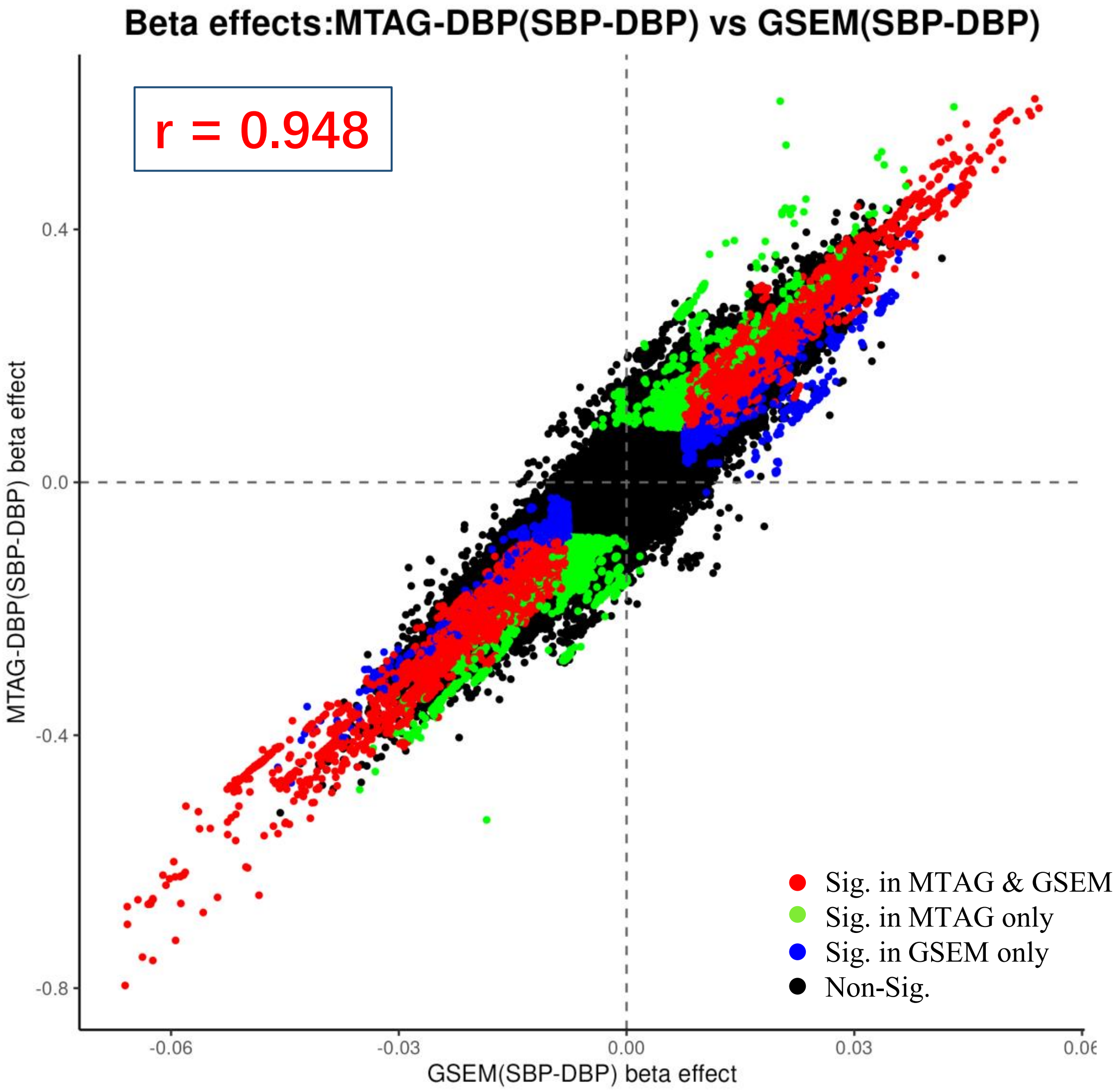
Beta effects:MTAG-DBP(SBP-DBP) vs GSEM(SBP-DBP)
r = 0.948
MTAG-DBP(SBP-DBP) beta effect
GSEM(SBP-DBP) beta effect
0.4
0.0
-0.4
-0.8
-0.06
-0.03
0.00
0.03
0.06
Sig. in MTAG & GSEM
Sig. in MTAG only
Sig. in GSEM only
Non-Sig.

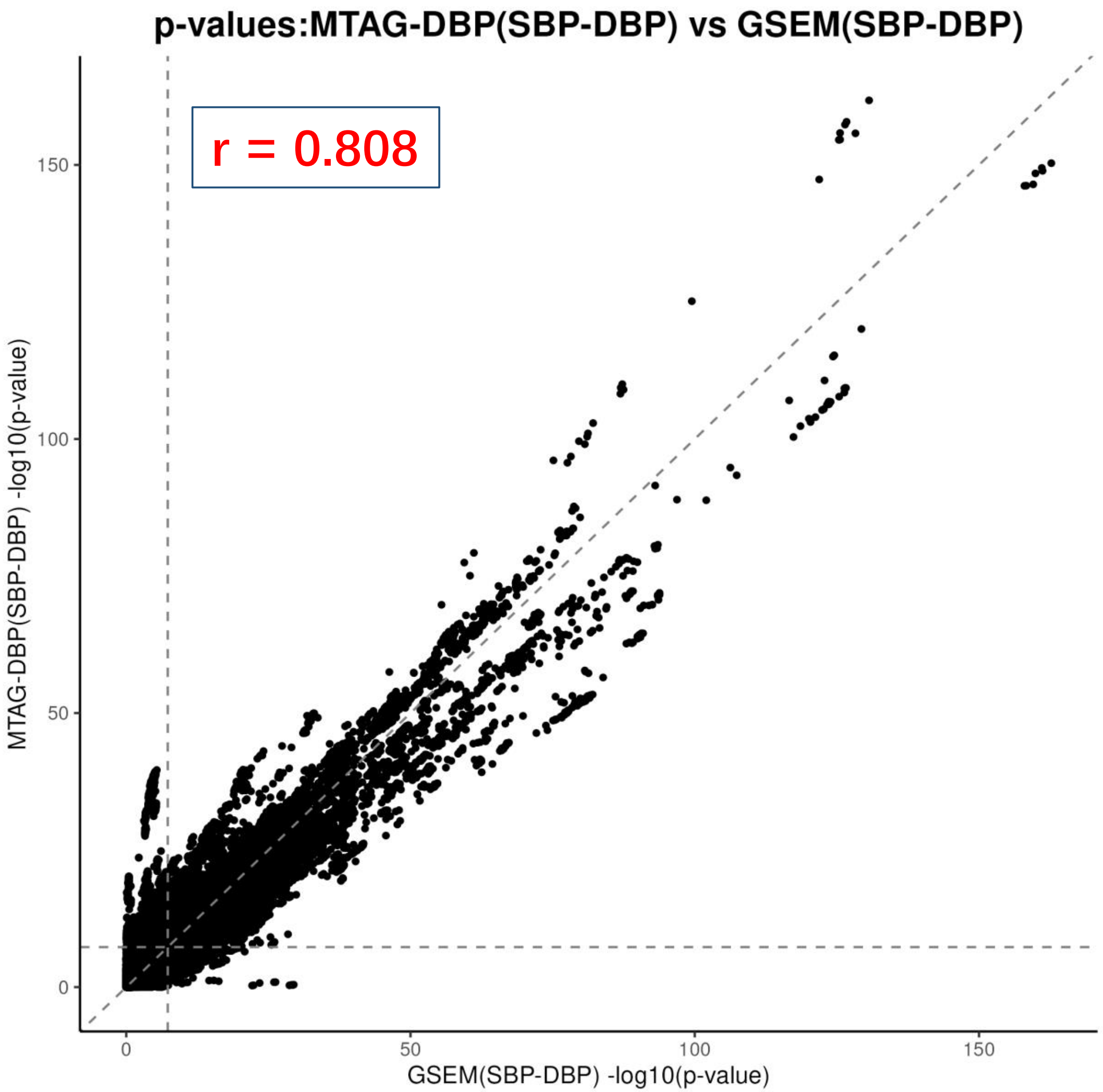
p-values:MTAG-DBP(SBP-DBP) vs GSEM(SBP-DBP)
r = 0.808
MTAG-DBP(SBP-DBP) -log10(p-value)
GSEM(SBP-DBP) -log10(p-value)
150
100
50
0
0
50
100
150

Sup Fig 1
(SBP-PP model)

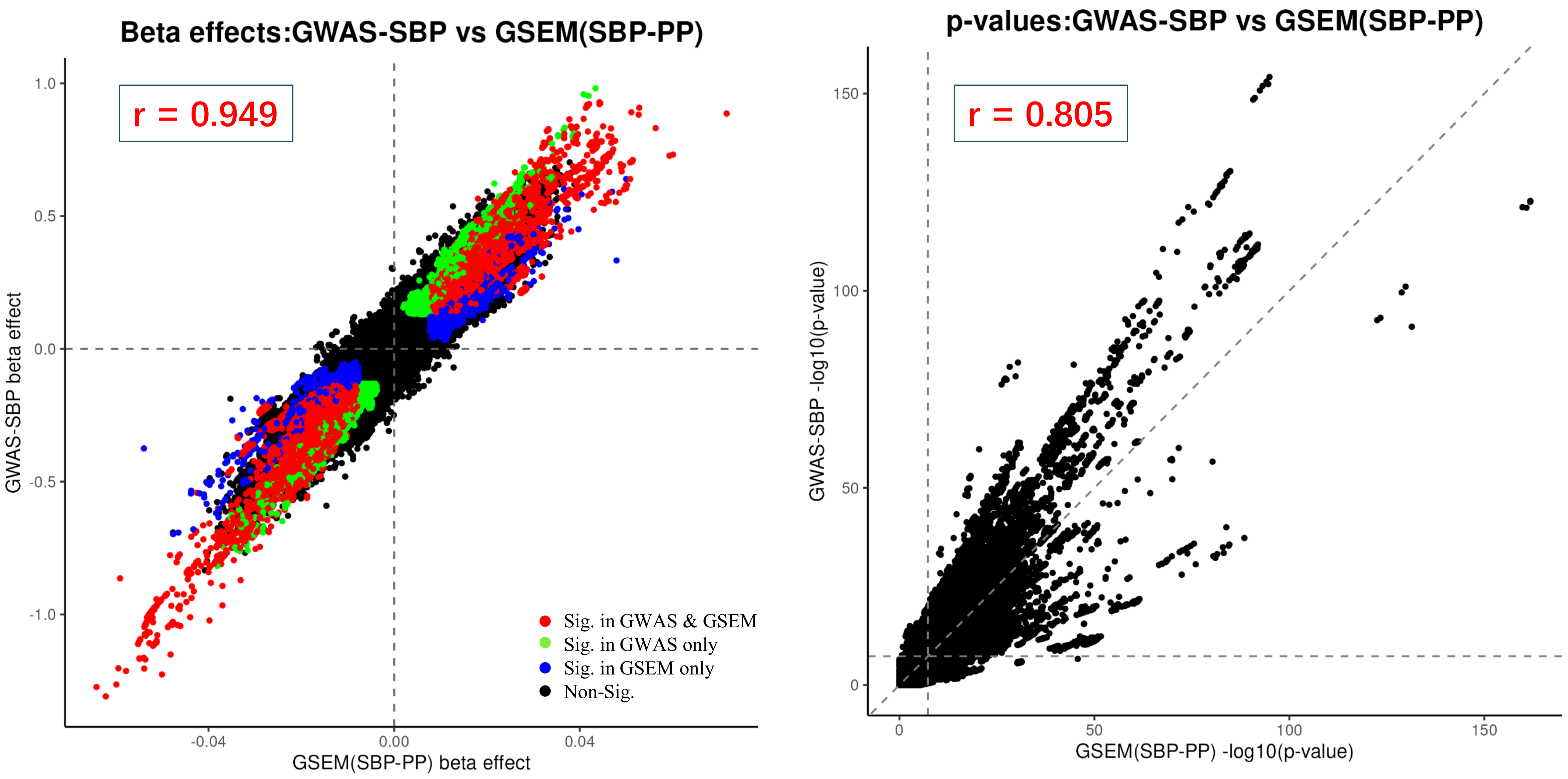

Beta effects:GWAS-SBP vs GSEM(SBP-PP)
r = 0.949
GWAS-SBP beta effect
1.0
0.5
0.0
-0.5
-1.0
-0.04
0.00
0.04
GSEM(SBP-PP) beta effect
Sig. in GWAS & GSEM
Sig. in GWAS only
Sig. in GSEM only
Non-Sig.
p-values:GWAS-SBP vs GSEM(SBP-PP)
r = 0.805
GWAS-SBP -log10(p-value)
150
100
50
0
0
50
100
150
GSEM(SBP-PP) -log10(p-value)

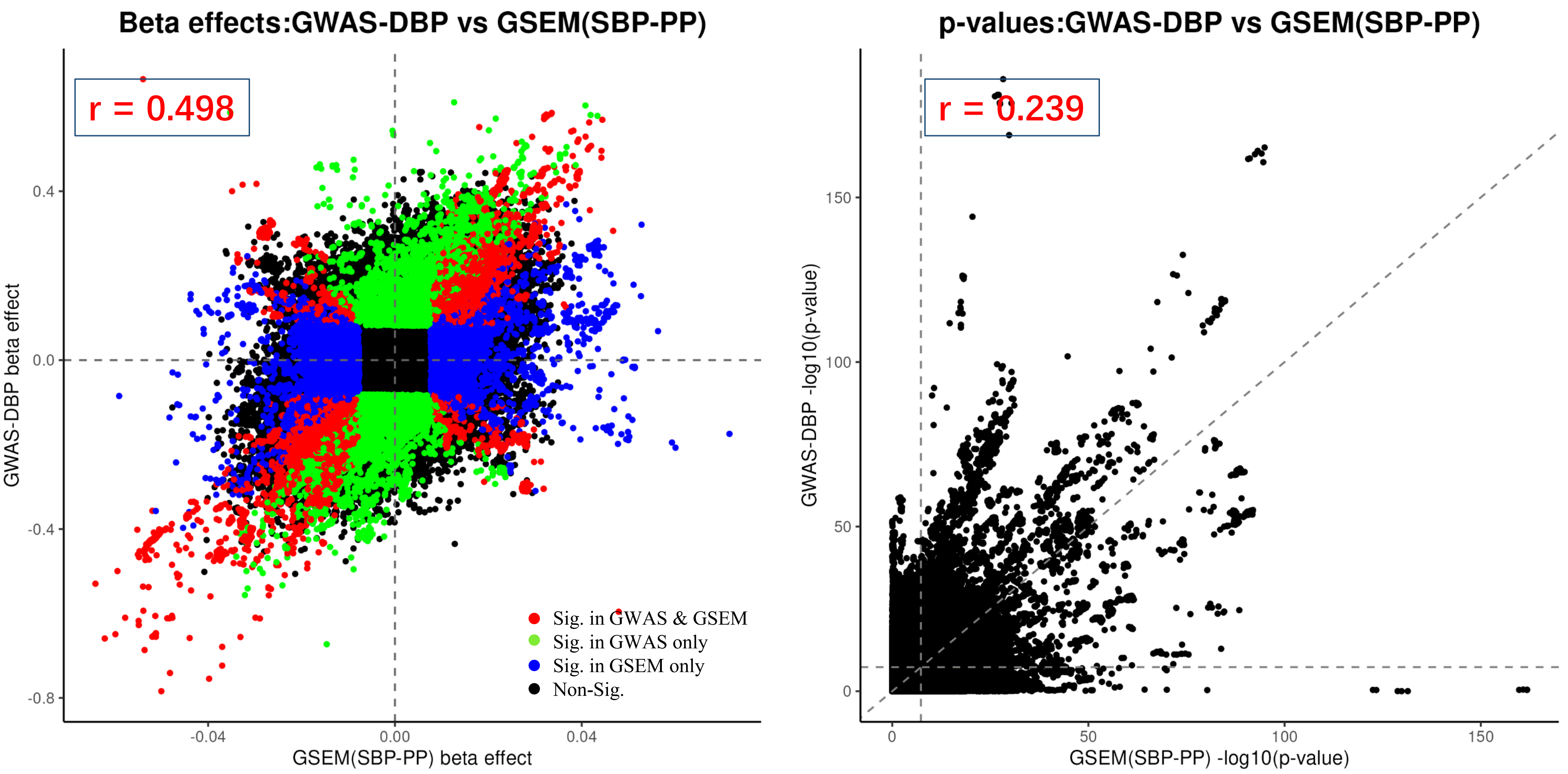
Beta effects:GWAS-DBP vs GSEM(SBP-PP)
r = 0.498
GWAS-DBP beta effect
GSEM(SBP-PP) beta effect
0.4
0.0
-0.4
-0.8
-0.04
0.00
0.04
Sig. in GWAS & GSEM
Sig. in GWAS only
Sig. in GSEM only
Non-Sig.
p-values:GWAS-DBP vs GSEM(SBP-PP)
r = 0.239
GWAS-DBP -log10(p-value)
GSEM(SBP-PP) -log10(p-value)
150
100
50
0
0
50
100
150

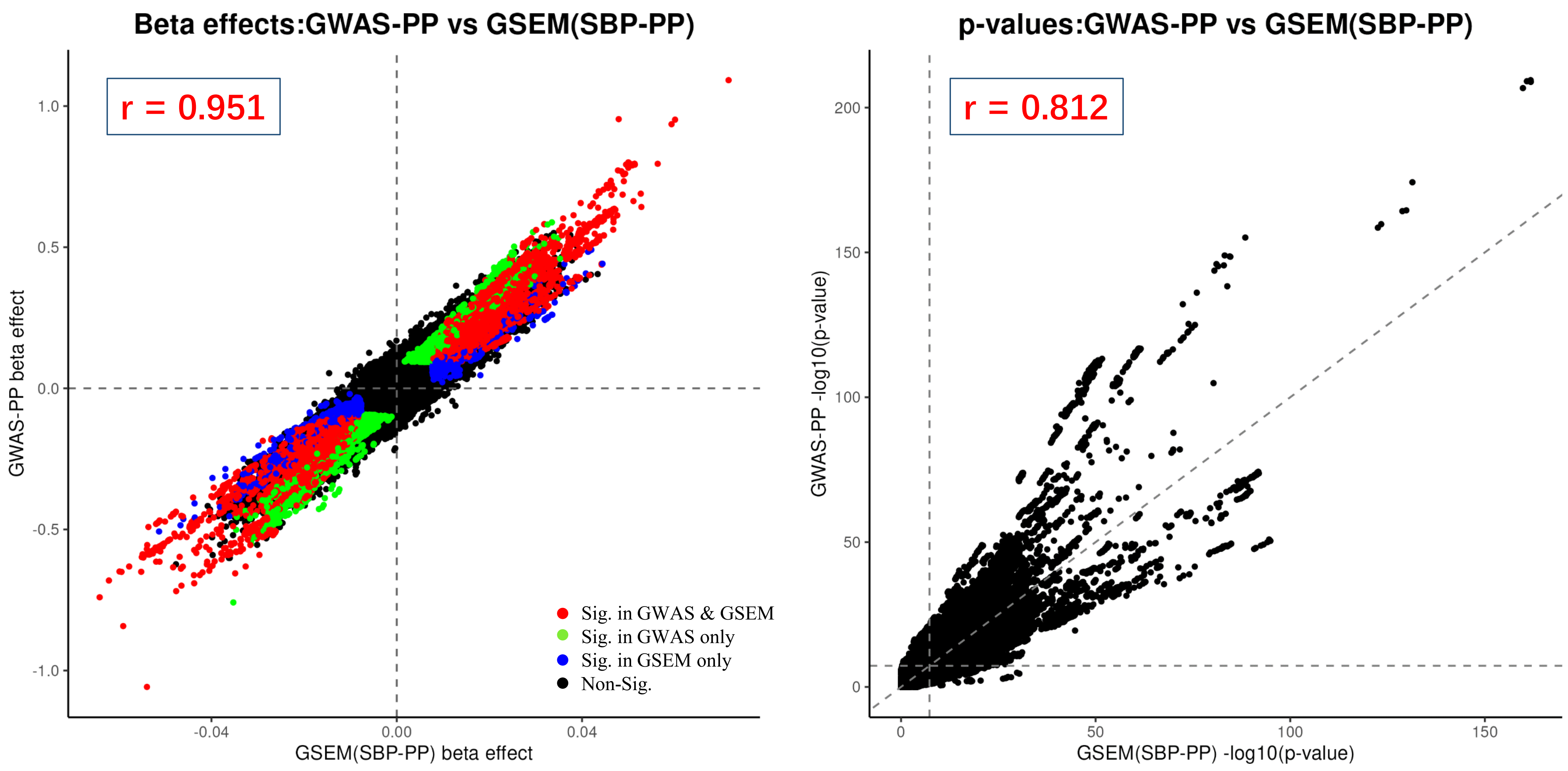
Beta effects:GWAS-PP vs GSEM(SBP-PP)
r = 0.951
GWAS-PP beta effect
GSEM(SBP-PP) beta effect
1.0
0.5
0.0
-0.5
-1.0
-0.04
0.00
0.04
Sig. in GWAS & GSEM
Sig. in GWAS only
Sig. in GSEM only
Non-Sig.
p-values:GWAS-PP vs GSEM(SBP-PP)
r = 0.812
GWAS-PP -log10(p-value)
GSEM(SBP-PP) -log10(p-value)
200
150
100
50
0
0
50
100
150

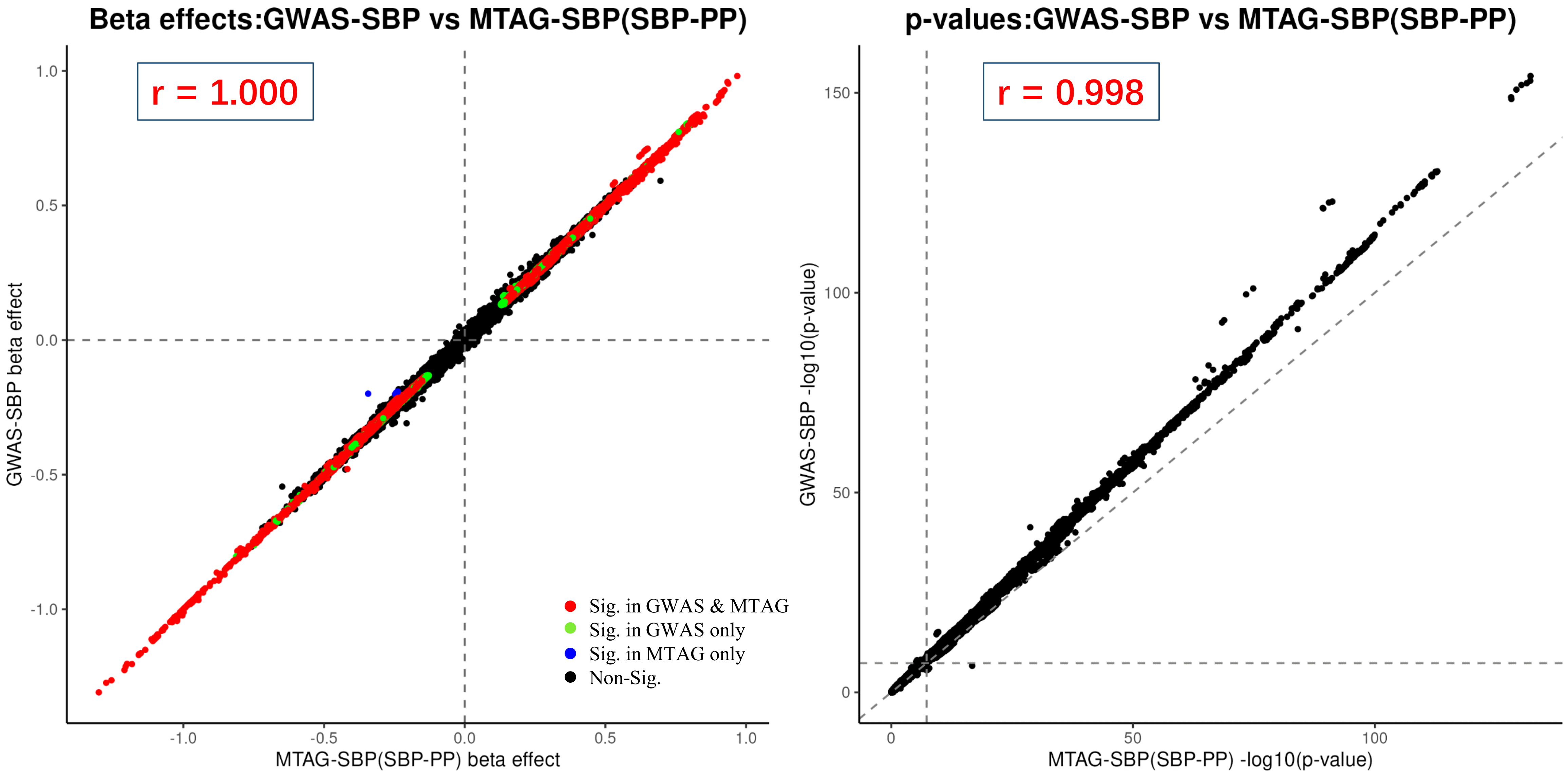
Beta effects:GWAS-SBP vs MTAG-SBP(SBP-PP)
r = 1.000
GWAS-SBP beta effect
MTAG-SBP(SBP-PP) beta effect
1.0
0.5
0.0
-0.5
-1.0
-1.0
-0.5
0.0
0.5
1.0
Sig. in GWAS & MTAG
Sig. in GWAS only
Sig. in MTAG only
Non-Sig.
p-values:GWAS-SBP vs MTAG-SBP(SBP-PP)
r = 0.998
GWAS-SBP -log10(p-value)
MTAG-SBP(SBP-PP) -log10(p-value)
150
100
50
0
0
50
100

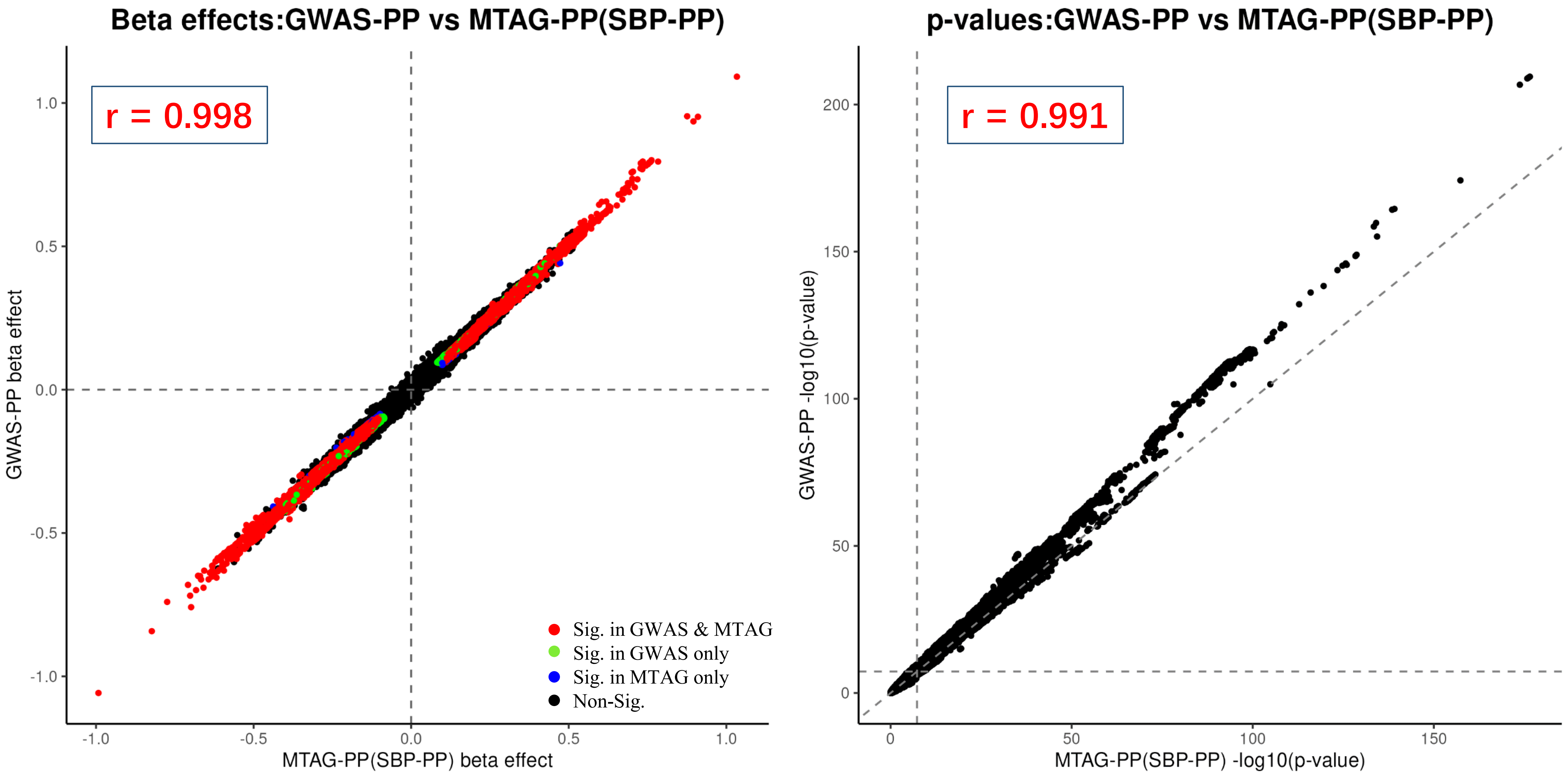
Beta effects:GWAS-PP vs MTAG-PP(SBP-PP)
r = 0.998
1.0
0.5
0.0
-0.5
-1.0
GWAS-PP beta effect
-1.0
-0.5
0.0
0.5
1.0
MTAG-PP(SBP-PP) beta effect
Sig. in GWAS & MTAG
Sig. in GWAS only
Sig. in MTAG only
Non-Sig.
p-values:GWAS-PP vs MTAG-PP(SBP-PP)
r = 0.991
200
150
100
50
0
GWAS-PP -log10(p-value)
0
50
100
150
MTAG-PP(SBP-PP) -log10(p-value)

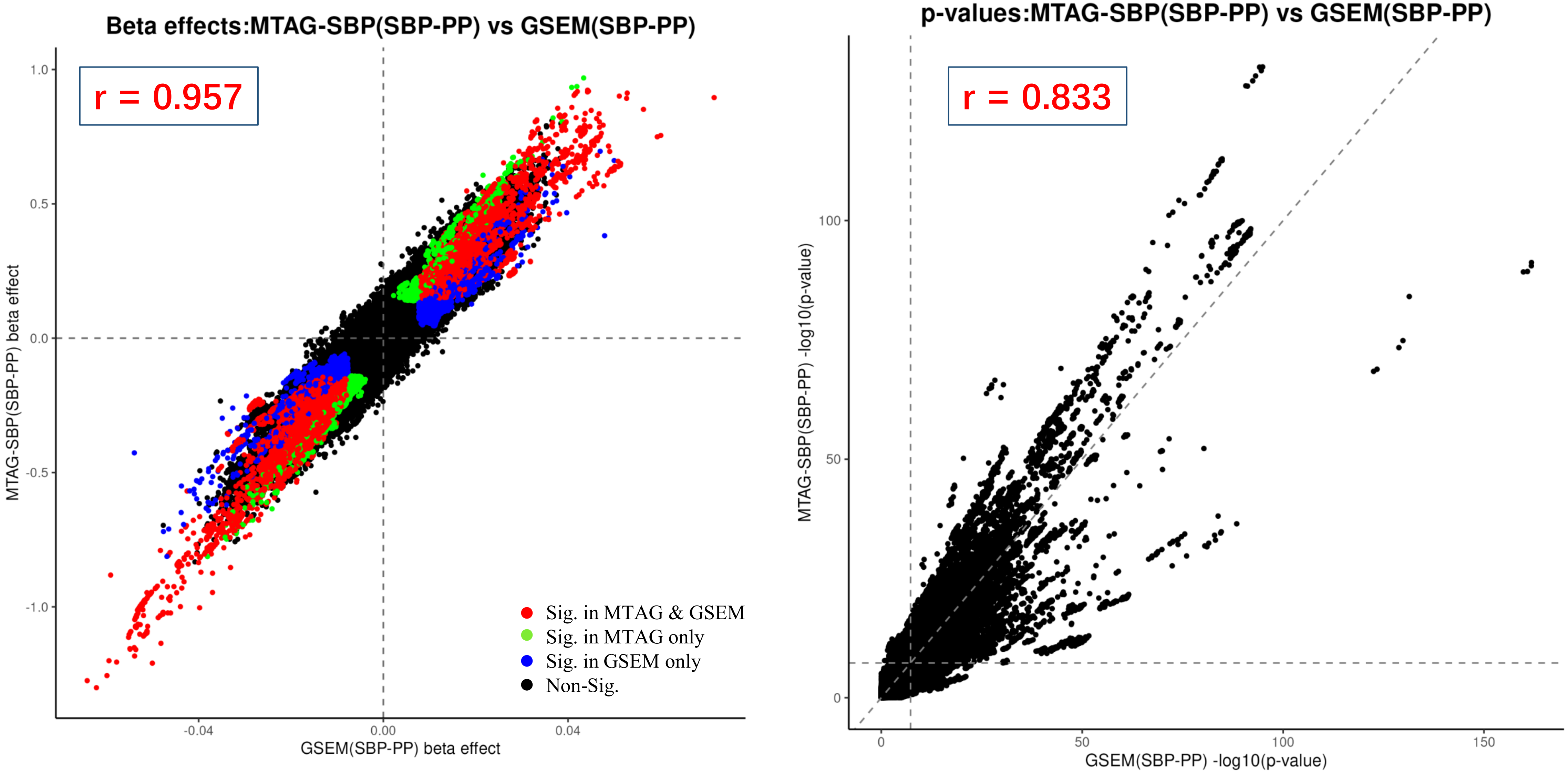
Beta effects:MTAG-SBP(SBP-PP) vs GSEM(SBP-PP)
r = 0.957
1.0
0.5
0.0
-0.5
-1.0
MTAG-SBP(SBP-PP) beta effect
-0.04
0.00
0.04
GSEM(SBP-PP) beta effect
Sig. in MTAG & GSEM
Sig. in MTAG only
Sig. in GSEM only
Non-Sig.
p-values:MTAG-SBP(SBP-PP) vs GSEM(SBP-PP)
r = 0.833
100
50
0
MTAG-SBP(SBP-PP) -log10(p-value)
0
50
100
150
GSEM(SBP-PP) -log10(p-value)

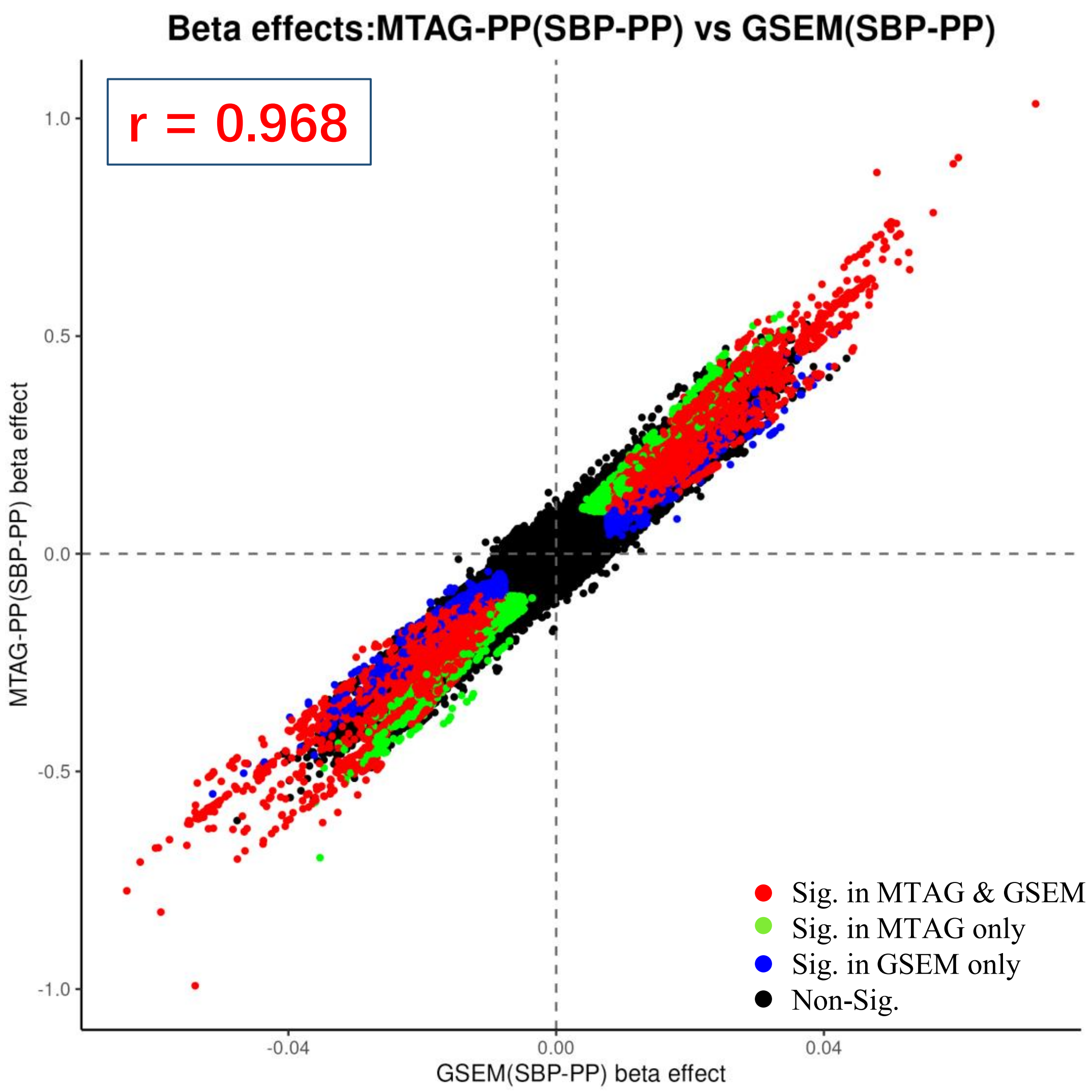
Beta effects:MTAG-PP(SBP-PP) vs GSEM(SBP-PP)
r = 0.968
1.0
0.5
0.0
-0.5
-1.0
MTAG-PP(SBP-PP) beta effect
-0.04
0.00
0.04
GSEM(SBP-PP) beta effect
Sig. in MTAG & GSEM
Sig. in MTAG only
Sig. in GSEM only
Non-Sig.

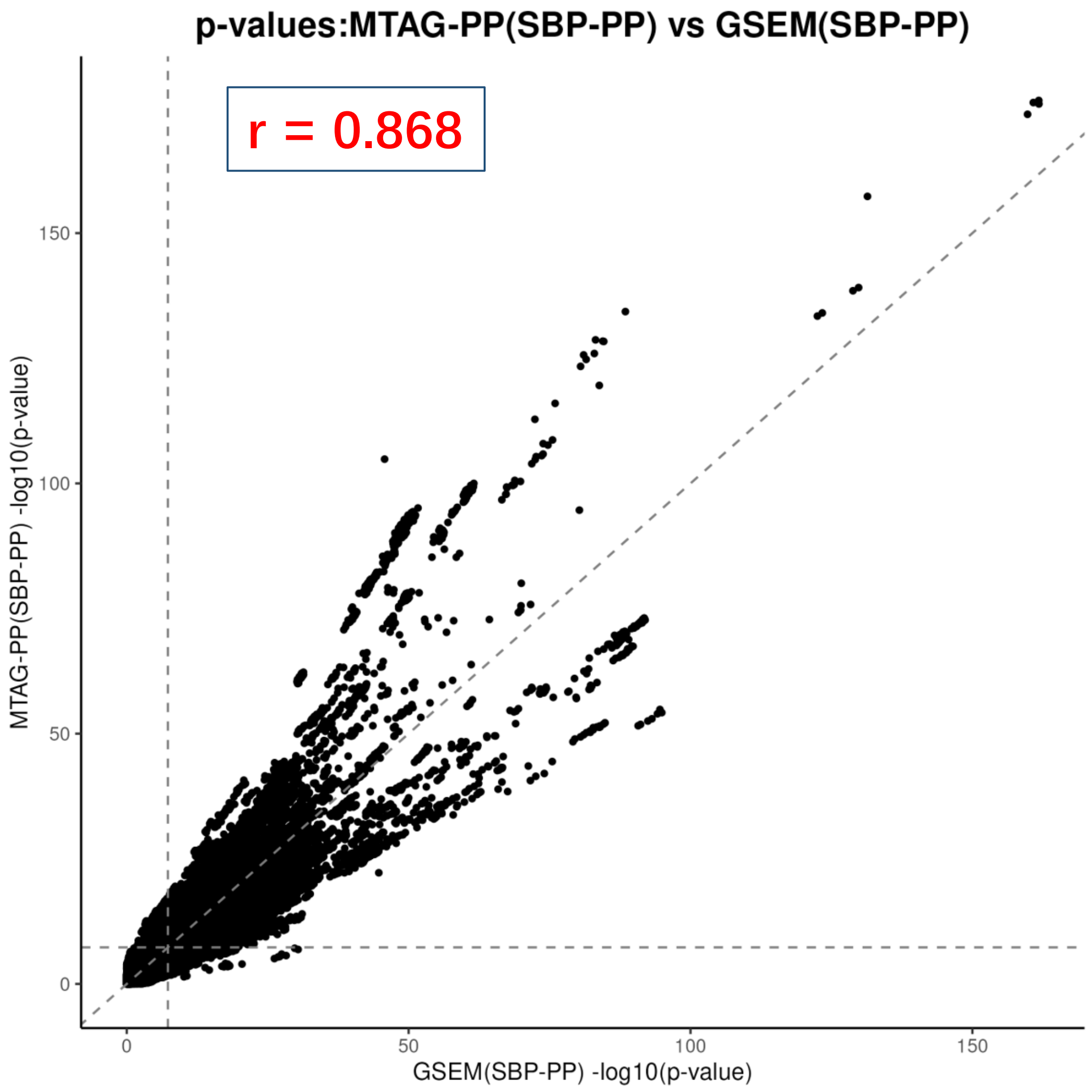
p-values:MTAG-PP(SBP-PP) vs GSEM(SBP-PP)
r = 0.868
150
100
50
0
MTAG-PP(SBP-PP) -log10(p-value)
0
50
100
150
GSEM(SBP-PP) -log10(p-value)

Sup Fig 1
(DBP-PP model)

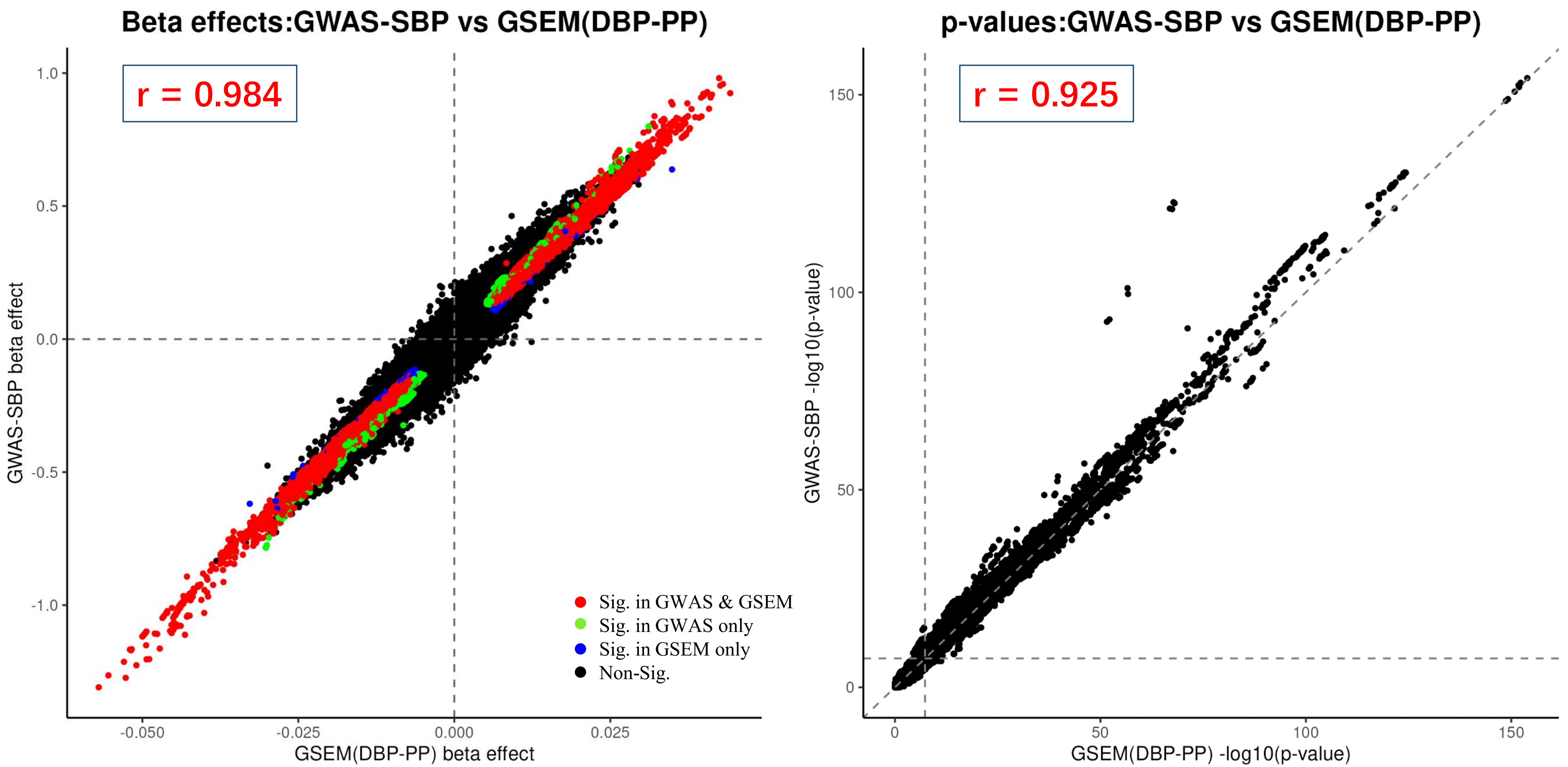
Beta effects:GWAS-SBP vs GSEM(DBP-PP)
r = 0.984
GWAS-SBP beta effect
GSEM(DBP-PP) beta effect
1.0
0.5
0.0
-0.5
-1.0
-0.050
-0.025
0.000
0.025
Sig. in GWAS & GSEM
Sig. in GWAS only
Sig. in GSEM only
Non-Sig.
p-values:GWAS-SBP vs GSEM(DBP-PP)
r = 0.925
GWAS-SBP -log10(p-value)
GSEM(DBP-PP) -log10(p-value)
150
100
50
0

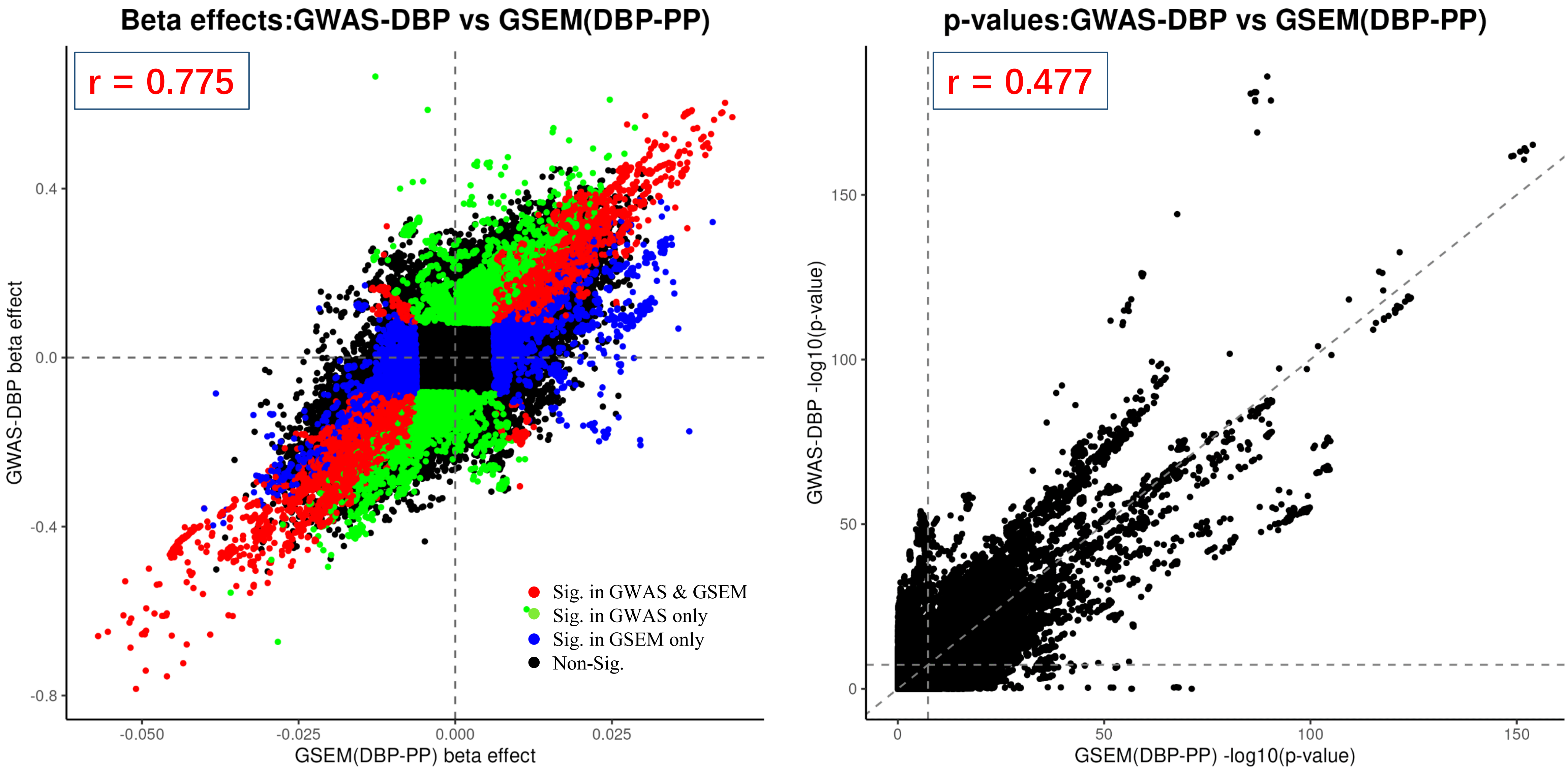

Beta effects:GWAS-DBP vs GSEM(DBP-PP)
r = 0.775
0.4
0.0
-0.4
-0.8
GWAS-DBP beta effect
-0.050
-0.025
0.000
0.025
GSEM(DBP-PP) beta effect
Sig. in GWAS & GSEM
Sig. in GWAS only
Sig. in GSEM only
Non-Sig.
p-values:GWAS-DBP vs GSEM(DBP-PP)
r = 0.477
150
100
50
0
GWAS-DBP -log10(p-value)
0
50
100
150
GSEM(DBP-PP) -log10(p-value)

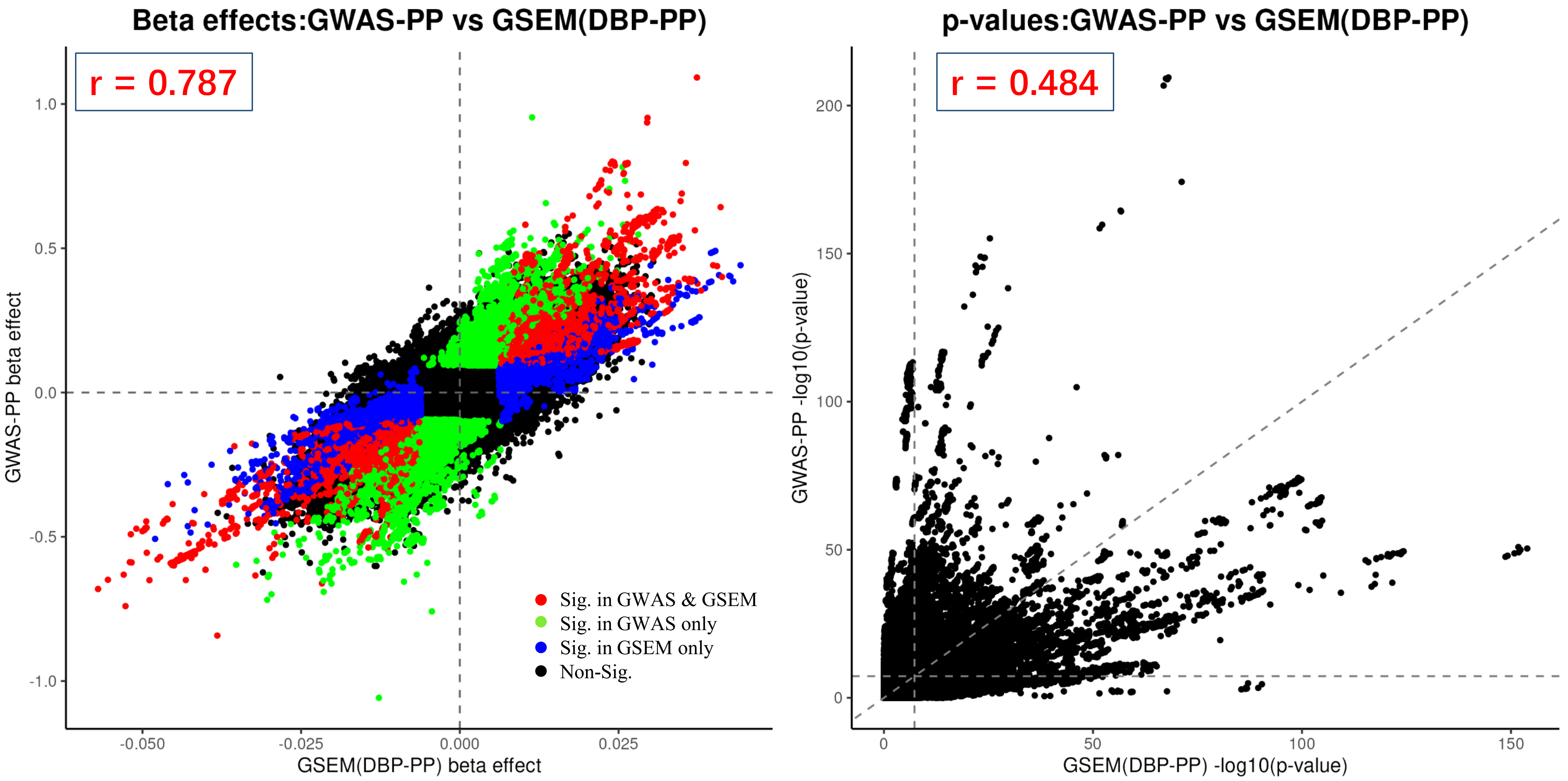

Beta effects:GWAS-PP vs GSEM(DBP-PP)
r = 0.787
GWAS-PP beta effect
GSEM(DBP-PP) beta effect
1.0
0.5
0.0
-0.5
-1.0
-0.050
-0.025
0.000
0.025
Sig. in GWAS & GSEM
Sig. in GWAS only
Sig. in GSEM only
Non-Sig.
p-values:GWAS-PP vs GSEM(DBP-PP)
r = 0.484
GWAS-PP -log10(p-value)
GSEM(DBP-PP) -log10(p-value)
200
150
100
50
0
0
50
100
150

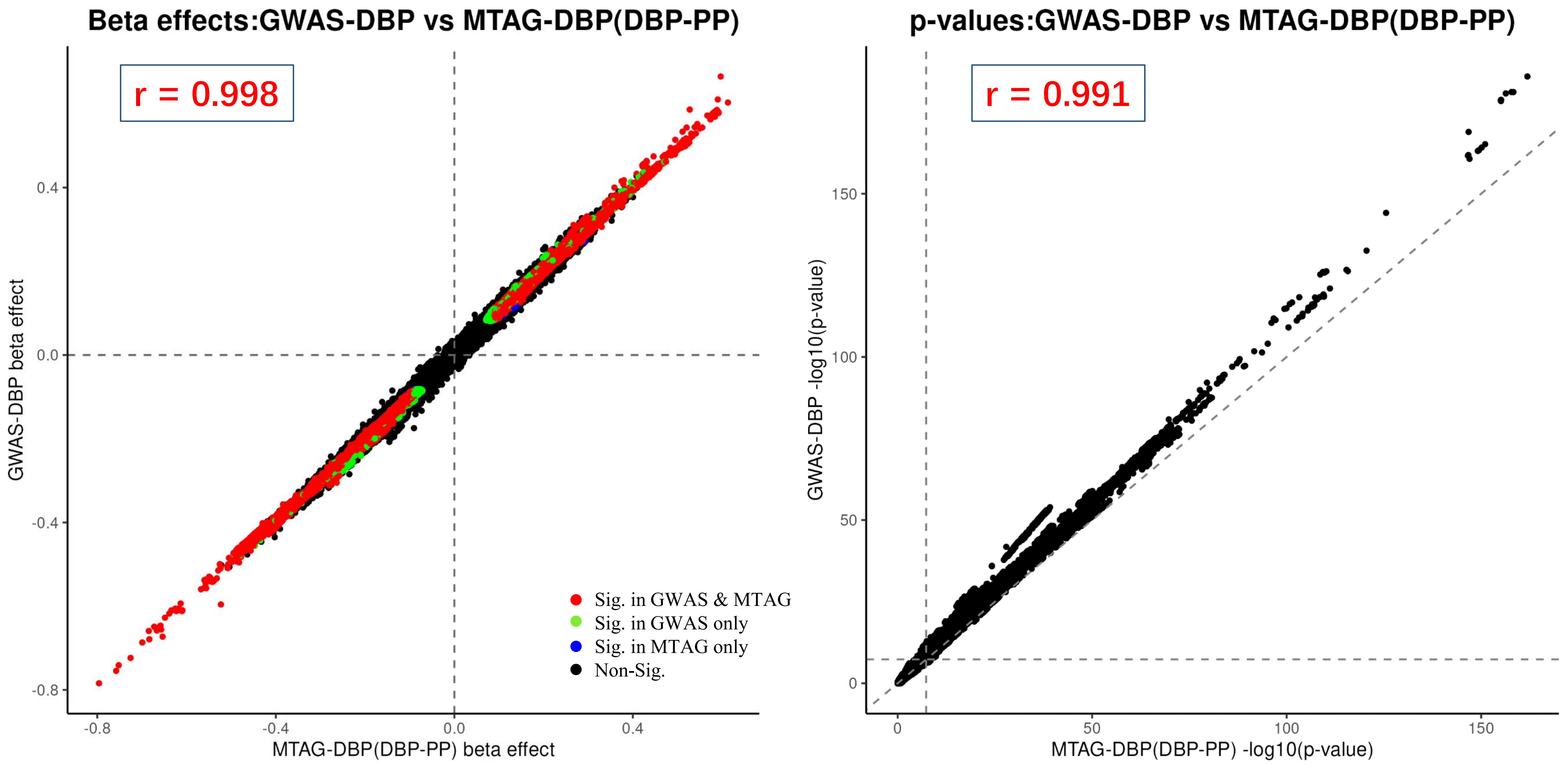
Beta effects:GWAS-DBP vs MTAG-DBP(DBP-PP)
r = 0.998
GWAS-DBP beta effect
MTAG-DBP(DBP-PP) beta effect
0.4
0.0
-0.4
-0.8
-0.8
-0.4
0.0
0.4
Sig. in GWAS & MTAG
Sig. in GWAS only
Sig. in MTAG only
Non-Sig.
p-values:GWAS-DBP vs MTAG-DBP(DBP-PP)
r = 0.991
GWAS-DBP -log10(p-value)
MTAG-DBP(DBP-PP) -log10(p-value)
150
100
50
0
0
50
100
150

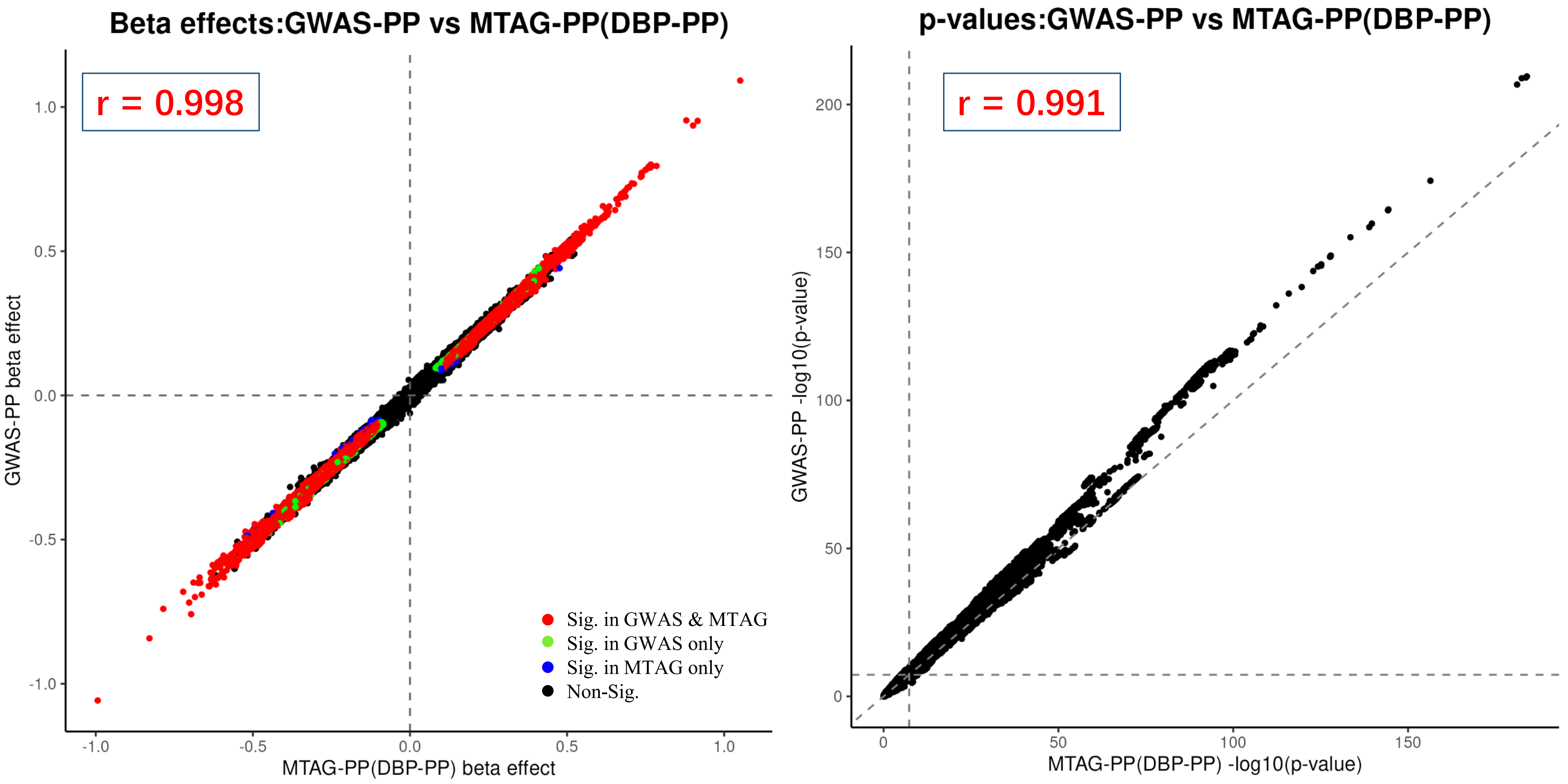
Beta effects:GWAS-PP vs MTAG-PP(DBP-PP)
r = 0.998
GWAS-PP beta effect
MTAG-PP(DBP-PP) beta effect
1.0
0.5
0.0
-0.5
-1.0
Sig. in GWAS & MTAG
Sig. in GWAS only
Sig. in MTAG only
Non-Sig.
p-values:GWAS-PP vs MTAG-PP(DBP-PP)
r = 0.991
GWAS-PP -log10(p-value)
MTAG-PP(DBP-PP) -log10(p-value)
200
150
100
50
0

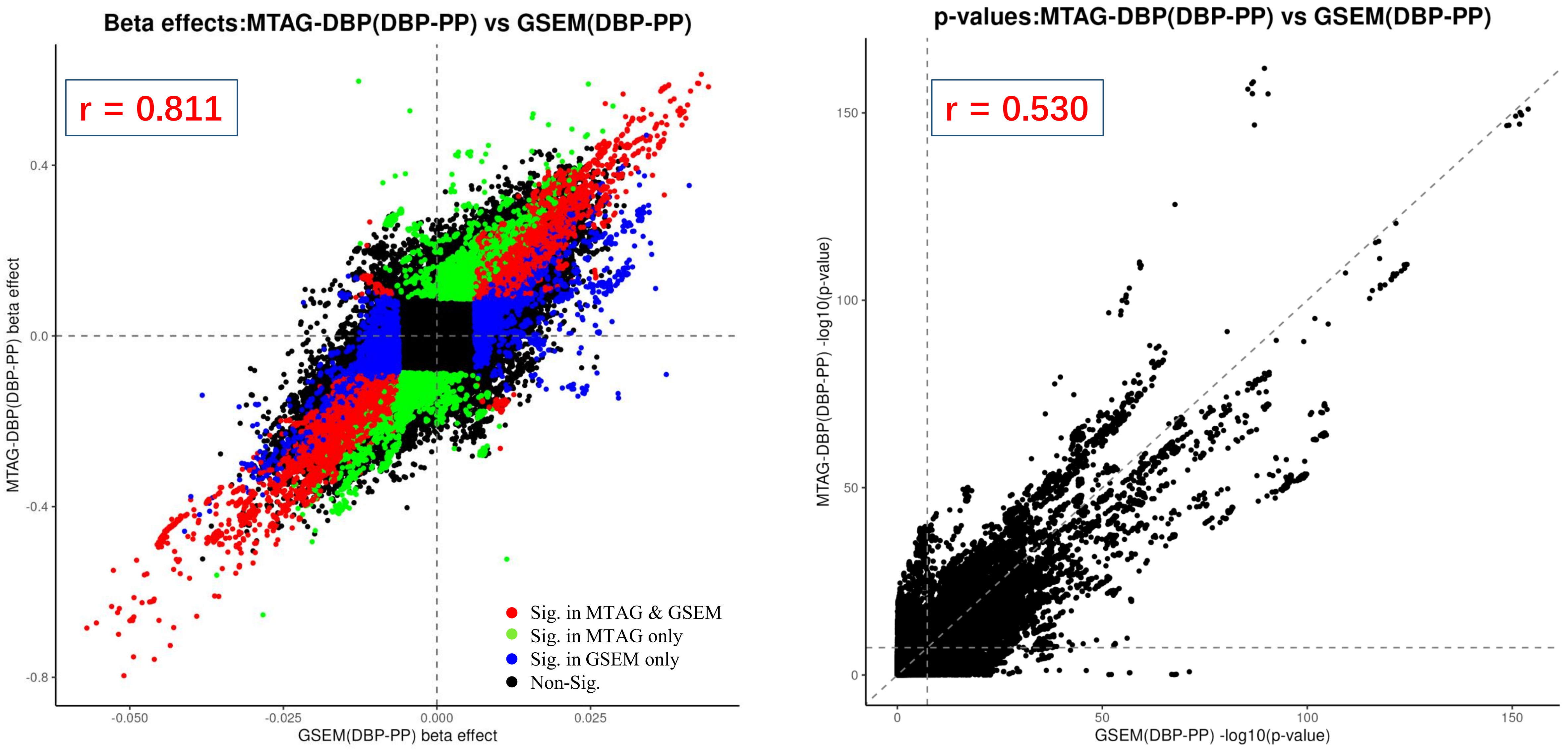
Beta effects:MTAG-DBP(DBP-PP) vs GSEM(DBP-PP)
r = 0.811
MTAG-DBP(DBP-PP) beta effect
GSEM(DBP-PP) beta effect
0.4
0.0
-0.4
-0.8
-0.050
-0.025
0.000
0.025
Sig. in MTAG & GSEM
Sig. in MTAG only
Sig. in GSEM only
Non-Sig.
p-values:MTAG-DBP(DBP-PP) vs GSEM(DBP-PP)
r = 0.530
MTAG-DBP(DBP-PP) -log10(p-value)
GSEM(DBP-PP) -log10(p-value)
150
100
50
0

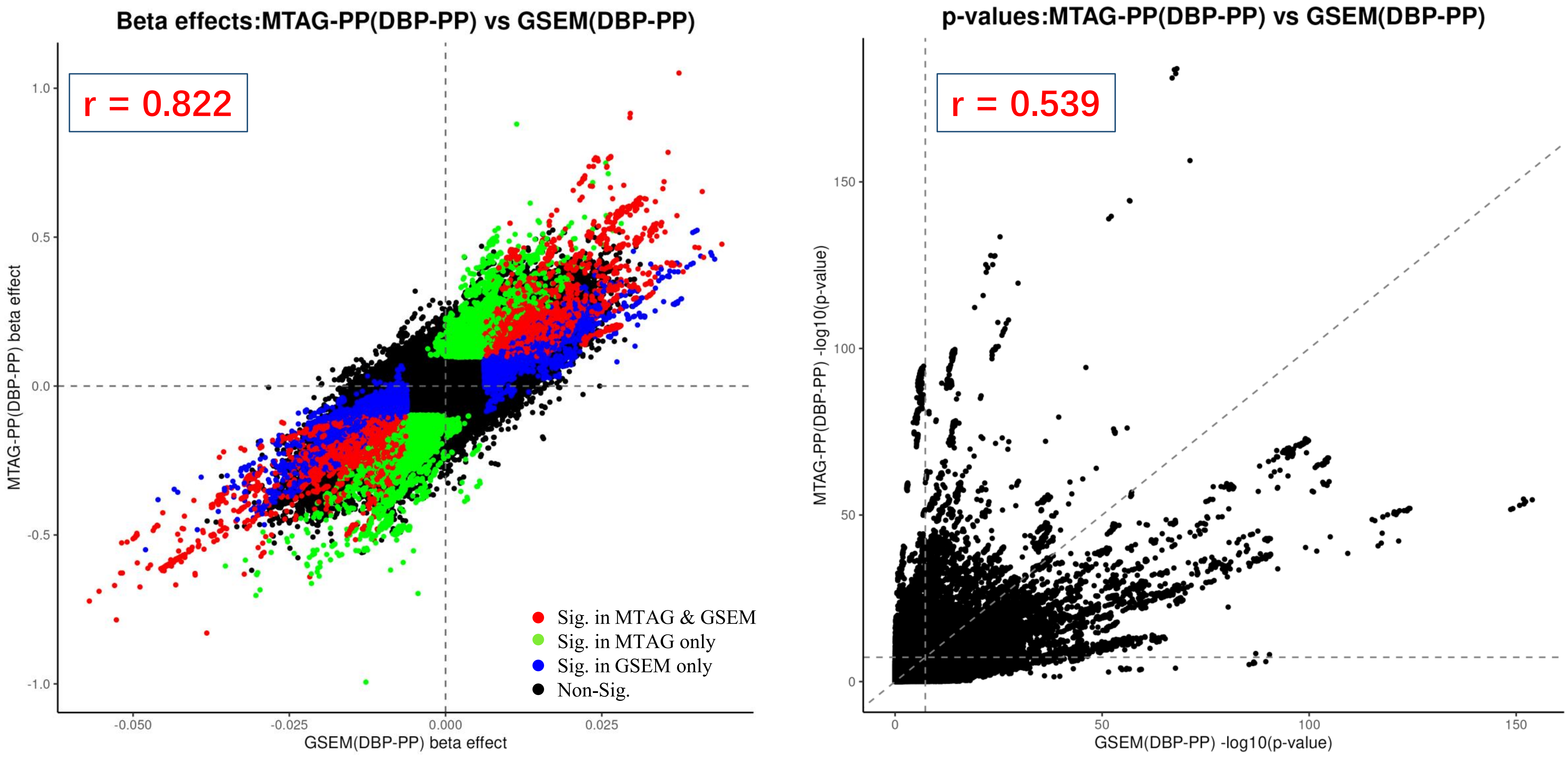

Beta effects:MTAG-PP(DBP-PP) vs GSEM(DBP-PP)
r = 0.822
MTAG-PP(DBP-PP) beta effect
GSEM(DBP-PP) beta effect
1.0
0.5
0.0
-0.5
-1.0
-0.050
-0.025
0.000
0.025
Sig. in MTAG & GSEM
Sig. in MTAG only
Sig. in GSEM only
Non-Sig.
p-values:MTAG-PP(DBP-PP) vs GSEM(DBP-PP)
r = 0.539
MTAG-PP(DBP-PP) -log10(p-value)
GSEM(DBP-PP) -log10(p-value)
150
100
50
0
0
50
100
150

Sup Fig 2

Locus Zoom Plot of Novel Locus

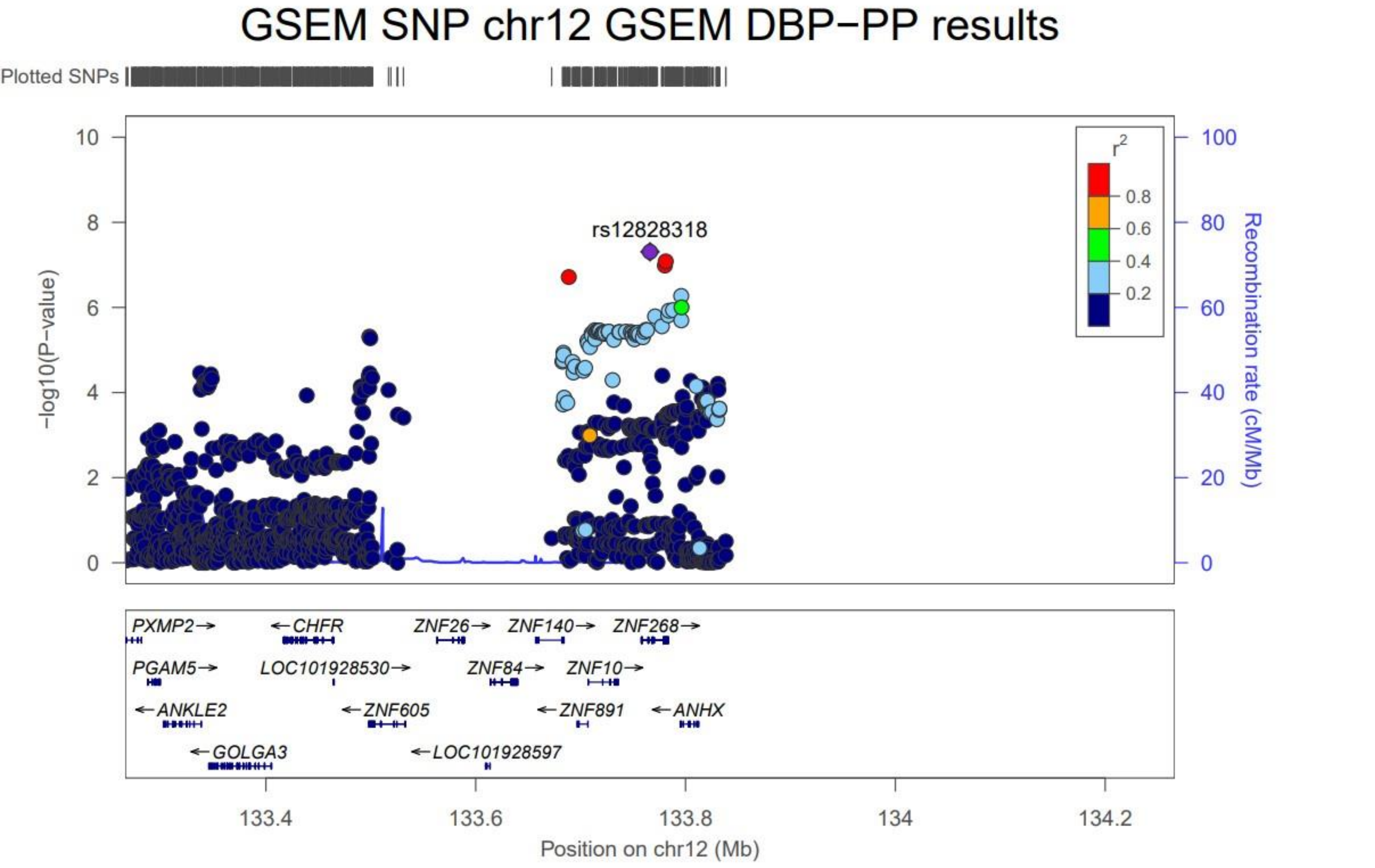
GSEM SNP chr12 GSEM DBP−PP results
Plotted SNPs
rs12828318
-log10(P-value)
Recombination rate (cM/Mb)
r2
0.8
0.6
0.4
0.2
PXMP2→
←CHFR
ZNF26→
ZNF140→
ZNF268→
PGAM5→
LOC101928530→
ZNF84→
ZNF10→
←ANKLE2
←ZNF605
←ZNF891
←ANHX
←GOLGA3
←LOC101928597
133.4
133.6
133.8
134
134.2
Position on chr12 (Mb)
